# Criteria for the prioritization of public health interventions for climate-sensitive vector-borne diseases in Quebec

**DOI:** 10.1371/journal.pone.0190049

**Published:** 2017-12-27

**Authors:** Valerie Hongoh, Pierre Gosselin, Pascal Michel, André Ravel, Jean-Philippe Waaub, Céline Campagna, Karim Samoura

**Affiliations:** 1 The Research Group on Epidemiology of Zoonoses and Public Health (GREZOSP), Faculty of Veterinary Medicine, Université de Montréal, Saint-Hyacinthe, Canada; 2 Department of Pathology and Microbiology, Faculty of Veterinary Medicine, Université de Montréal, Saint-Hyacinthe, Canada; 3 Institut national de santé publique Québec (INSPQ), Québec, Canada; 4 Ouranos, Consortium on Regional Climatology and Adaptation to Climate Change, Montreal, Canada; 5 National Microbiology Laboratory at Saint-Hyacinthe, Public Health Agency of Canada, Saint-Hyacinthe, Canada; 6 Group for Research in Decision Analysis (GERAD, HEC Montréal, Polytechnique Montréal, McGill, UQAM), Montreal, Canada; 7 Department of Social and Preventive Medicine, Université Laval, Québec, Canada; 8 Université Aube Nouvelle, Ouagadougou, Burkina Faso; University of Washington, UNITED STATES

## Abstract

Prioritizing resources for optimal responses to an ever growing list of existing and emerging infectious diseases represents an important challenge to public health. In the context of climate change, there is increasing anticipated variability in the occurrence of infectious diseases, notably climate-sensitive vector-borne diseases. An essential step in prioritizing efforts is to identify what considerations and concerns to take into account to guide decisions and thus set disease priorities. This study was designed to perform a comprehensive review of criteria for vector-borne disease prioritization, assess their applicability in a context of climate change with a diverse cross-section of stakeholders in order to produce a baseline list of considerations to use in this decision-making context. Differences in stakeholder choices were examined with regards to prioritization of these criteria for research, surveillance and disease prevention and control objectives. A preliminary list of criteria was identified following a review of the literature. Discussions with stakeholders were held to consolidate and validate this list of criteria and examine their effects on disease prioritization. After this validation phase, a total of 21 criteria were retained. A pilot vector-borne disease prioritization exercise was conducted using PROMETHEE to examine the effects of the retained criteria on prioritization in different intervention domains. Overall, concerns expressed by stakeholders for prioritization were well aligned with categories of criteria identified in previous prioritization studies. Weighting by category was consistent between stakeholders overall, though some significant differences were found between public health and non-public health stakeholders. From this exercise, a general model for climate-sensitive vector-borne disease prioritization has been developed that can be used as a starting point for further public health prioritization exercises relating to research, surveillance, and prevention and control interventions in a context of climate change. Multi-stakeholder engagement in prioritization can help broaden the range of criteria taken into account, offer opportunities for early identification of potential challenges and may facilitate acceptability of any resulting decisions.

## Introduction

Prioritizing resources for optimal response to an ever growing list of existing and emerging infectious disease risks presents an important challenge to public health administrations and their core intervention domains [1,2]. Ongoing global changes such as climate change, large scale land use transformations, increasing global travel and political instability in various regions of the world, contribute to variations in the patterns and occurrence of a number of infectious diseases, notably vector-borne diseases, which are known to be sensitive to weather and climate [3]. Changes in terms of the season of occurrence and the geographical distribution of these diseases are anticipated to increase as weather and climate are known to be drivers of the transmission and distribution of vector-borne diseases [4]. Prioritizing resources between existing and climate sensitive vector-borne diseases is complex but a necessary reality as potentially difficult trade-offs need to be made while taking into account a diversity of viewpoints [5]. For example, how much should be invested in surveillance activities, including vector surveillance and laboratory testing of novel pathogens, for diseases that are not yet endemic in Canada when resources for surveillance of existing diseases are limited? In other words, what non-endemic diseases should we be conducting surveillance for? Adaptation to vector-borne diseases will require both incremental and transformational approaches [6]. This study examines prioritization of climate-sensitive vector-borne diseases as an incremental step in improving management of these diseases within the primary domains of public health activity: research, surveillance, and prevention & control. Prioritization often serves as an initial step in aligning efforts and guiding public health decisions. In Quebec, MCDA methods have been used by the National Public health Institute and the Ministry of health for the prioritisation of Lyme disease surveillance and West nile virus interventions. The prioritization exercises referred to in this study pertain to planning of activities over a 1-5-year time scale while recognizing that these exercises may need to be updated and revisited within that time frame as knowledge evolves. The interventions included in these three domains range from the search for new treatments and diagnostic methods to education and outreach interventions as well as field interventions such as vector control or modifications to the built environment to help reduce the human health impact of infectious diseases. Several disease prioritization exercises have been undertaken in public health and veterinary public health contexts over the last few decades [7–21]. Traditionally, where stakeholders have been involved in these processes, with the exception of the studies by Ng and Sargeant (2012a) and Brookes et al (2014b), experts in public health have been the primary stakeholders included in the process. Prioritization exercises assist in explicitly bringing into focus the issues of concern around the decision problem and systematically evaluating available evidence on these issues. In this way, these exercises help structure reflection and guide decisions around resource allocation in order to ensure effectiveness within organisations and across various levels of government for effective public health delivery [22]. In other words, what are the concerns, what is the state of knowledge around these concerns, what are options going forward and how do these options perform in light of stated concerns. Prioritization exercises have been carried out at various scales from institutional-level [10], country-level [16,23–29] to continent-level [30,31]. Depending on the context, these exercises have been carried out either by institutional representative groups, a cross-section of the expert community concerned, advisory groups, national officials or researchers. Concern over how to prioritize infectious diseases in a context of climate change is more recent and at the the time of the initial review, had been documented only in the prioritization exercise by Cox and colleagues (2013). An evolution in the way prioritization exercises have been carried out over the last few decades can be seen in the examination of the literature [5] and reveals common goals and concerns that have persisted in their undertaking; notably, a push towards a systematic and transparent process and growing awareness of the increasing viewpoints that should be included in such exercises [13,22,25,30]. Refinements to the disease prioritization process over time have sought to separate information on the diseases (criteria measurement) from values pertaining to prioritization concerns (criteria) in order to improve transparency of the process. The explicit and measurable aspect of these exercises is sought by defining explicit criteria on which to evaluate the diseases being prioritized. For example, the measure of whether a disease is increasing or decreasing in the population can be captured as a criterion on the current incidence of human cases in the country; however, other concerns must also be evaluated such as what is the severity of the disease, do effective treatments already exist to limit the disease, is the general population already aware of and adopting effective protective behaviour to prevent the disease? If a disease is increasing in the population but not a severe disease or perhaps effective treatment already exists, should we allocate additional resources to this disease? For instance, Lyme disease is currently increasing in Canada, but relatively effective antibiotic treatment exists, how should this disease be prioritized relative to a new disease not yet endemic to Canada for which no treatment exists such as Dengue? Does the priority of a disease differ whether we are discussing allocation of resources in a research context versus a surveillance or prevention and control context? Criteria and how they are used to evaluate diseases are at the crux of the disease prioritization process. Criteria should represent core considerations or values relating to the prioritization objectives and help explicitly track relative differences between the items being prioritized [32]. Additionally, since health decisions in publicly funded health care systems use tax-paying citizen’s dollars to operate, in the interest of transparency and accountability, it is important to understand what concerns are held by society both to verify acceptability of potential decisions made following prioritization exercises and understand where differences in values or priorities may be present in order to help bridge existing gaps. For example, does society feel that resources should be prioritized for diseases that have more severe effects on vulnerable groups such as the elderly, very young, or pregnant women versus less severe diseases that affect a greater number of individuals? Examining the impact of different prioritization methodologies on the resulting prioritized list of diseases is also an important issue to explore.

In the current study, we identify criteria for the prioritization of vector-borne diseases applicable in a context of climate change in order to construct a general model for disease prioritization. We then examine differences in concerns expressed by stakeholders with regards to prioritization of public health interventions relating to research, surveillance and prevention and control objectives. Stakeholders working in fields both directly and not directly connected to public health were included in the process to broaden the diversity of perspectives and voices considered in the disease prioritization process, as well as assess similarities and differences in values held. While the inclusion of stakeholders from outside of public health is not yet routine in public health decision making, exercises have been conducted to assess the acceptability and ground feasibility of proposed interventions [33], as well as level of public awareness on a subject, such as the level of Lyme disease awareness in Canada [34], which may be useful in guiding future allocation of resources towards educational outreach activities.

The effect of combining current scientific knowledge with stakeholder values on disease prioritization is examined here by means of a pilot prioritization exercise performed with a multicriteria decision aid (MCDA) process using the PROMETHEE (Preference Ranking Organization Method for Enrichment Evaluations) outranking method [35].

## Materials and methods

**Fig 1:**
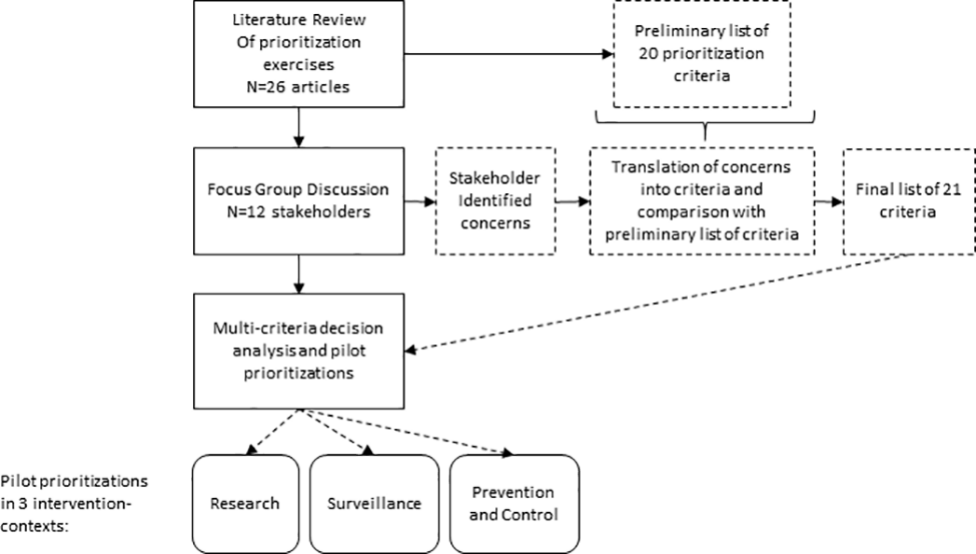
Flow chart of steps conducted to create disease prioritization models.

In disease prioritization exercises, criteria are used to systematically take into account concerns relevant to the decision-making context. The use of a participatory multi-stakeholder processes contributes to a broader and transparent selection of decision criteria. Here, a comprehensive review of the disease prioritization literature was conducted to create a synthesis list of the most commonly used prioritization criteria relevant in a context of climate change. These were discussed and validated with a group of stakeholders. The resulting criteria were used in a pilot prioritization exercise using PROMETHEE to examine differences in stakeholder assigned weights and their effect on prioritization under different intervention domains (research, surveillance, prevention and control). These steps are discussed in further details in the following sections and illustrated in a flow chart in Fig 1.

### Preliminary criteria identification

A comprehensive review of infectious disease prioritization studies published between 1990 and 2014 was undertaken in Spring 2014 to identify key criteria that should be considered for inclusion in a generic model (Table 1). A keyword search of the literature was carried out using a scientific database (Pubmed) with combinations of the following keywords: “emerging”, “infectious”, “communicable”, “zoonoses”, “disease” and “prioritization”. Titles and abstracts were used to identify potentially relevant articles for further data extraction. Articles published in English or French pertaining to a prioritization exercise of infectious disease related items were retained for review. Additionally, relevant peer reviewed and grey literature referenced by articles retained for data extraction were also included in the review if they met the original inclusion criteria (snowball sampling) [36]. Relevant criteria and their related categories as described by citing articles were extracted from reviewed studies. As climate change may alter the season of occurrence and geographical distribution of climate sensitive vector-borne diseases [4,6], criteria pertaining to elements of the disease transmission process that may be affected by climate change were included. Whether or not conditions for transmission are already present for a disease is an important consideration in prioritization of these diseases. Conditions for disease emergence and transmission in a context of climate change can relate to environmental conditions, vector or reservoir conditions. For example, *Anopheles* species of mosquito capable of transmitting malaria are present in Canada and thus represent a vector component necessary for endemic transmission of this disease in Canada. Climate change signals are inherently difficult to separate out from the multitude of other driving forces (such as land use patterns, globalization and associated transport of goods, travel, etc.) which may affect disease transmission patterns. As such, many criteria that pertain to general infectious disease prioritization, such as the underlying vulnerability and susceptibility of the population to a disease or current levels of scientific knowledge and treatment availability, are also relevant in assessing climate sensitive vector-borne diseases and therefore were included in the preliminary list. Criteria were organized into categories following the literature review to facilitate criteria discussion with stakeholders and is presented along with the criteria in the results section.

**Table 1:** Article selection process for review.

	Steps	Total articles
1	Initial keyword search in Pubmed of studies containing combinations of the following keywords: “emerging”, “infectious”, “communicable”, “zoonotic”, “disease” and “prioritization”.	N = 1196
2	Title and abstract scan of articles from Step 1 scanned for relevance resulting in 37 studies describing describing disease prioritization studies.	N = 37
3	Related peer reviewed and grey literature articles referenced by articles retained in step 2 were also scanned for relevance (snowball search).	N = 42
4	Final article selection of studies in which prioritization criteria were explicitly listed or described	N = 26*

*Note: In some cases, multiple articles referred to different aspects of the same study

### Focus group discussion

Following the literature review, a focus group discussion was held with a small group of stakeholders in Quebec (Canada) in September 2014 to discuss concerns with regards to vector-borne diseases in a context of climate change. Prioritization of diseases was further examined in the context of interventions for research, surveillance as well as prevention and control. Stakeholder invitations were initially sent out to 24 individuals and organizations having previously participated in vector-borne disease consultations by the province. These organizations represent a range of civil, municipal and regional level organizations (including non-governmental environmental rights groups, Sustainable Development, Environment, Wildlife and Parks of Quebec, the Ministry of Agriculture, Fisheries and Food (of Quebec), the Council for the Protection of Sick Patients (of Quebec), municipal representatives from municipalities in the province, representatives from the Health and Welfare Commissioner (of Quebec), academic researchers, representatives from a Quebec seniors rights representative group, regional public health departments and provincial blood donation services) representing the interests of various civil, municipal or regional subsets of the Quebec population. These organizations have all been in existence for over a decade. Stakeholders were selected based on their availability for a concurrent participation in a separate ongoing study on West Nile virus management in Quebec. Implications of this selection are discussed in further detail in the discussion section of this paper. All participating stakeholders gave informed written consent prior to participation in the study. Prior to the stakeholder meeting, participants were invited to reflect on their concerns with regards to managing infectious diseases now and over the course of the next 5 to 10 years in the context of climate change. For example, are diseases with high severity of greater concern than diseases with high incidence or vice versa? What other concerns are of interest, economic concerns, logistic concerns such as treatment availability or knowledge levels of the disease, animal and environmental health impact related concerns? During the focus group discussion, stakeholders began by writing down their prepared concerns. These were then compiled using a modified nominal group technique [37] and discussed with all participants. Following this, the literature identified criteria and their categories were presented in writing and discussed with stakeholders. Stakeholder compiled concerns and literature identified criteria were compared and discussed. Stakeholders were given the opportunity to add additional criteria and clarify or reword the literature identified set in person during the discussion. Measurement scales were also discussed at this time. Following the meeting, stakeholders were given an additional month to reflect on and validate the final list of criteria by means of two rounds of online Delphi review [38]. The online review was conducted using an electronic survey that presented all retained criteria from the in-person discussion and allowed stakeholders to “vote” for the inclusion of individual criteria. Comments on the relevance of the criteria to the prioritization models could also be made at this time. Stakeholders agreed to retain any criteria which received at least one vote from a participating stakeholder. Results from the first online review were compiled and presented to stakeholders to allow further modification before the final validated set was defined. This final set was included in prioritization models pertaining to research, surveillance and prevention and control of vector-borne diseases. This project was reviewed and approved by the Ethical Committee for Health Research of the University of Montreal (Comité d’éthique de la recherche en santé, CERES) (certificate number 14-025-CERES-D).

### Criteria weighting

Following validation of the final list of criteria, stakeholders were asked to weight criteria according to their relative importance with regards to research, surveillance and prevention and control objectives. The purpose of this weighting exercise was to translate stakeholder value systems into numerical weights. In order to do this, stakeholders were given a Microsoft Excel spreadsheet tool and asked to distribute 100 points across the list of decision criteria included in the model. The Excel tool included the finalized list of stakeholder validated criteria, desired effect direction of criteria and measurement scales listed by category with replicated sections for each of the three intervention domains (research, surveillance, prevention and control). The desired effect direction of a criterion refers to what the desired state is with respect to that criterion. For instance, with respect to costs, the desired effect direction is generally to prioritize diseases with the highest cost burden, i.e. maximize this criterion; whereas with respect to the general level of knowledge of the public on a disease, the desired effect direction may be to prioritize diseases that the public has little knowledge of, i.e. minimize this criterion. Stakeholders were asked to weight criteria in accordance with perceived importance taking into account their relative importance overall. Weights of zero were permitted for criteria to allow stakeholders to indicate the absence of importance of criteria if applicable. The difference in relative weights assigned to different categories were compared between the three intervention domains (research, surveillance and prevention and control) and Welch’s *t*-test (unequal variances *t*-test) were performed in R (version 3.2.2) (R Core Team (2016), Vienna, Austria, http://www.R-project.org) to test for significant differences in category weights.

### Pilot prioritization of five diseases

An exploratory prioritization of five potentially climate-sensitive vector-borne diseases, Lyme, West Nile virus, chikungunya, dengue, and malaria was carried out to examine the effects of criteria weightings described in the previous section on disease rankings for each intervention domain. Only Lyme and West Nile virus have shown a local transmission cycle in Quebec in the last 10 years [39,40]; the other three diseases currently manifest themselves as imported cases only, but local cycles may occur in the coming decades due to climate change [41–43]. Lyme expansion in North America has been linked to climate change [39] and while West Nile virus expansion into North America was not directly linked to climate change, its epidemiology has been shown to be directly sensitive to climatic factors [44]. A literature search was conducted in late fall 2014 in Pubmed using each of the five diseases separately and in combination with the 21 identified criteria in order to assess and score disease performance over the criteria. Only articles published in English or French pertaining to the disease and criteria were reviewed. In other words, given the latest literature, what evaluation does each disease obtain on each of the identified criteria using the specified measurement scale. The same disease assessment scores were used for each intervention domain, however, stakeholders weighted each intervention domain separately and weights were shown to vary depending on the domain. Analysis of disease performance and criteria weights was performed with the PROMETHEE method (Preference Ranking Organization Method for Enrichment Evaluations) in visual PROMETHEE software (version 1.4.0.0) (VP Solutions software, Brussels, Belgium, http://www.promethee-gaia.net). This MCDA method requires a set of criteria with corresponding weights and diseases performance scores over each of the criteria in order to produce a ranking of the relative performance of all diseases over the combined criteria. The PROMETHEE II method was used as it provides a complete ranking of results without incomparability [45,46]. PROMETHEE methods are pair-wise comparison methods that are part of the outranking class of decision aid methods enabling comparison of multiple items over multiple criteria [45,46]. In traditional multi-criteria type problems, incomparability can occur when one option performs better on certain criteria while another option performs better on very different criteria making comparison challenging [45,46]. PROMETHEE II was designed to overcome this incomparability without scale effects by calculating a net performance score over all criteria [45,46]. Additionally, a GAIA (Geometrical Analysis for Interactive Aid) visual analysis was also used to graphically explore decision maps. A sensitivity analysis was performed to examine the robustness of rankings for the 1^st^ order given weights expressed by stakeholders.

## Results

### Literature and stakeholder identified criteria

Following an initial keyword search, the titles and abstracts of 1196 articles were scanned for relevance (Table 1). This resulted in 37 studies which were retained for full text review. Five additional articles referenced within the previous set were also reviewed. From this, 26 studies explicitly reporting prioritization criteria were retained for data extraction. A summary of these studies is shown in Table 2. Studies were primarily from high income countries in North America, Europe and Asia. While prioritization exercises have taken place in developing contexts [47], none were found pertaining explicitly to vector-borne diseases in the reviewed time period. An initial list of 122 criteria was extracted from these studies. The number of criteria used per prioritization exercise ranged from as few as 5 to as many as 57. Reported sources included experts, and lists from previous exercises and the literature.

**Table 2:** Summary of reviewed disease prioritization studies.

Author & year	Country^	Objective	**	Criteria	Weights	#	Item type	Method overview
Carter 1991	Canada	Set priorities for national surveillance (notifiable list)	B	12	No	60	Communicable diseases	Committee (n = 6) scored and discussed. Un-weighted criteria. Cut-off set for inclusion of diseases on notifiable list.
Rushdy et al 1998	UK	Rank diseases to manage resources	C	6	No	41	33 communicable diseases and 8 generic diseases	Expert opinion, questionnaire—assessed by experts in communicable diseases (n = 194)
Doherty 2000	Canada	To inform resource allocation national level	B, D	10	No	43	Communicable diseases	Expert opinion and consensus of subcommittee (n = 6)
Horby et al 2001	UK	Rank diseases to manage resources	B, C	5	No	69	58 pathogens and 11 generic diseases	Expert opinion (n = 518)
Valenciano 2002 (InVS)	France	Determine priorities to improve knowledge, prevention and control of diseases	A, B, C	6	No	37	Non-food borne zoonoses	Expert opinion (n = 10)
WHO 2002 (Dubrovnik pledge) *	WHO—7 eastern European countries	Strengthen infectious disease surveillance systems in 7 countries of South-East Europe	B	8	No	53	Communicable diseases	Expert opinion (n = 24)
Doherty 2006	Canada	Strengthen national surveillance capacities	B	10	No	48	Communicable diseases	Expert opinion and consensus of subcommittee (n = 6)
McKenzie et al 2007	New Zealand	Prioritize wildlife pathogens for surveillance	B	3	No	82	Wildlife pathogens	OIE based risk assessment approach
Krause et al 2008a&b	Germany	Guide research and surveillance strategies of department	A,B	12	Yes	85	Pathogens	Expert opinion (n = 11) and weighted sum aggregation
Cardoen et al 2009	Belgium	Rank food and water-borne pathogens to prioritize resource allocation for management	C	5	Yes	51	Food and water-borne zoonotic pathogens	Expert opinion (n = 35) and weighted sum aggregation
Capek 2010 (InVS)*	France	Rank non-foodborne zoonoses and anticipate emerging threats linked to climate, etc.	A, B, C	6	No	37	Non-food borne zoonoses	Expert opinion (n = 16)
Havelaar et al 2010	The Netherlands	Prioritized emerging zoonoses to support an early warning and surveillance network	B	7	Yes	86	Emerging zoonotic pathogens	MCDA technique. Existing list and expert opinion determined list of pathogens, weighting of criteria based on panel consultation (n = 29)
Pavlin et al 2010	Pacific Island nations	Update list of pathogens to include on urgent NNDL list	B, D	12	Yes	27	Conditions/diseases assessed	Additive model—Sum of scores
Ruzante et al 2010	Canada	Framework to prioritize foodborne risks	D	4	Yes	6	Pathogen-food combinations	MCDA technique—PROMETHEE
Balabanova et al 2011	Germany	Rank infectious diseases for research and surveillance	B	10	Yes	127	Pathogens	Expert opinion (n = 83) and weighted sum aggregation
Humblet et al 2012	Europe	European collaboration and agreement on priority zoonoses for surveillance and eradication	B, C	57	Yes	100	Zoonoses	MCDA technique with Expert scoring (n = 40) with weighted sum aggregation and Monte Carlo simulation
Ng & Sargeant 2012a,b, 2013	Canada	Compare zoonoses priorities between Canada and the US from public and expert perspective	A	21 (59)†	Yes	62	Zoonotic diseases	Criteria elicitation—via conjoint analysis technique conducted with public (n = 1500) and expert (n = 1471) focus groups and surveys, summed using part-worth utility values approach
Cediel et al 2013	Colombia	Prioritize zoonoses for surveillance	B	12	Yes	32	Zoonoses	Delphi (n = 12) and additive model
Del Rio Vilas et al 2013	UK	To inform management of emerging animal health related threats in UK	C	10‡	Yes	111	111 threats, 74 unique	MCDA technique—Developed threat assessment tool
Cox et al 2013	Canada	Test standardised method to prioritise infectious diseases of humans and animals that may emerge in response to CC	A	40	Yes	9	Trialed on 9 test pathogens	MCDA technique—MACBETH and additive model (n = 64)
Kadohira et al 2015	Japan	Surveillance and management of zoonoses	B, C	7	Yes	98	Zoonoses	Author determined criteria, risk profiles generated and reviewed by experts (n = 76) with AHP attributed weights by stakeholder groups (n = 334)
Brookes et al 2014 a&b	Australia	Prioritize exotic pig diseases for management	C	9	Yes	30	Diseases	MCDA technique with stakeholder (n = 81) elicited weight preference via online survey

^ Country targeted by prioritization exercise

* Not peer reviewed

** A = research; B = surveillance; C = prevention & control; D = policy

†59 identified, but only 21 used in prioritization exercises

‡3 models (perception (3 criteria), impacts (4 criteria) and capabilities (3 criteria))

A number of studies had shared approaches and criteria (e.g.: [7,23,24]; [22,48]; [8,10,27,49]) while other studies used similar concepts, with variations in wording. The most common categories of criteria included: public health impacts, economic or market impacts, animal health impacts (generally pertaining to market impacts but also for animal-welfare), public perception and public health capacity to deal with a disease. These categories were retained in the models and named “Public Health”, “Social Impact”, “Economic”, “Animal and Environmental Health”, “Strategic and Operational” (i.e. logistics). Additionally, a “Risk and Epidemiology” category was added to capture epidemic potential, recent disease trends and proportion of susceptible population. Climate sensitive risk and epidemiology were also included in this category. As climate change will likely alter temperature and precipitation patterns with consequences for animal and vector distribution [4], criteria pertaining to existing conditions for disease transmission were included here. Commonly used prioritization criteria and their frequency were tracked across reviewed studies (see supplementary S1 Table). Recurring relevant criteria were identified and where wording was different but pertaining to the same concept, criteria were combined and synthesized where appropriate into a shorter preliminary list of 20 criteria covering as broad a range of relevant concepts as possible for discussion with stakeholders. This number was chosen in order to present a manageable set for discussion with stakeholders. Retained criteria were then used in a pilot prioritization exercise to examine differences in stakeholder assigned weights under different intervention domains.

### Focus group discussion

Twelve stakeholders consented to participate in a discussion held on September 29^th^, 2014 in Montreal, Quebec, Canada on the topic of perspectives and concerns relevant to disease prioritization in a context of climate change. One third of participants were female. All participants were between the ages of 30 and 65. Stakeholders had backgrounds in microbiology, entomology, biology, medicine, veterinary medicine and patient advocacy and hailed from a mix of both provincial and municipal organizations. Stakeholder discussions revealed coherence between stakeholder identified concerns and the literature constructed list of criteria. Further online validation by stakeholders following the initial meeting, resulted in a finalized list of twenty-one criteria (Table 3). Discussions of appropriate measurement scales and direction of desired effect to assess diseases were also held and are included in Table 3.

**Table 3:** Stakeholder validated list of criteria for the prioritization of climate sensitive vector-borne diseases.

Category	Criteria	Effect direction	Measurement units
Public Health Criteria (PHC)	PHC-01 –Reported yearly incidence of human cases in country	Maximize	0: Nil; 1: Very Low (<5); 2: Low (6–30); 3: Moderate (31-; 100): High (101–500); 5: very high (>500); 6: Unknown
	PHC-02 –Severity of the disease (both physically and mentally)	Maximize	0: Nil; 1: Low severity; 2: Moderate severity; 3: High severity; 4: Very high severity (risk of mortality)
	PHC-03 –Vulnerable groups	Maximize	0: All are vulnerable; 1: Existence of higher risk groups (e.g. 0-5yrs)
	PHC-04 –Potential to increase social inequality *	Maximize	0: No effect on social inequality; 1: Likely to exacerbate social inequality
Social Impact Criteria (SIC)	SIC-01 –Risk perception of the public	Maximize	1: Low perceived importance; 2: Moderate importance; 3: High importance
	SIC-02 –General level of knowledge, attitude and behaviour of the public	Minimize (Diseases for which the public has little knowledge of greater concern)	1: Little or no knowledge; 2: Moderate knowledge (general idea of symptoms); 3: High knowledge (can recognize symptoms and aware of transmission and treatment)
Risk and Epidemiology Criteria (REC)	REC-01 –Existence of favourable conditions for disease transmission	Maximize (diseases for which transmission conditions already favourable of greater concern)	1: Low risk (climate not suitable, no vector and no reservoir hosts); 2: Moderate risk (one of components present, either suitable climate, vector or reservoir host); 3: High risk (all components present–suitable climate, vector and reservoir host—or current or historic transmission)
	REC-02 –Epidemic potential	Maximize	1: Low risk; 2: high risk
	REC-03 –Current global trend of disease over last 5 years	Maximize	1: Stable–little to no recent local or global change in transmission; 2: unstable–recent global changes in transmission; 3: very unstable–recent local changes in transmission
	REC-04 –Proportion of susceptible population	Maximize	1: very low 0–5%; 2: low 5–10%; 3: moderate 10–25%; 4: high 25–50%; 5: very high 50+
Animal and Environmental Health Criteria (AEC)	AEC-01 –Estimated prevalence of yearly animal cases	Maximize (diseases with more cases of greater concern)	0: not transmissible to animals; 1: very low (<5%); 2: low (5–10%); 3: moderate (10–25%); 4: high (25–50%); 5: very high (50+); 6: unknown prevalence
	AEC-02 –Severity of disease	Maximize	0: Not applicable; 1: Low severity; 2: Moderate severity; 3: High severity; 4: Very high severity (risk of mortality)
	AEC-03 –Environmental or animal reservoir stage	Maximize (diseases with environmental stages of greater concern; harder to control)	1: Low risk–no independent stages that can survive in environment, water or reservoir hosts; 2: higher risk–existence of independent stages that can survive in environment, water or reservoir hosts.
Economic Criteria (ECC)	ECC-01 –Cost to provincial government	Maximize	1: low costs; (a few thousand); 2: moderate costs (hundreds of thousands); 3: high costs (millions)
	ECC-02 –Cost to private sector	Maximize	1: low costs (<100$); 2: moderate costs (<1000$); 3: high costs (>1000$)
	ECC-03 –Cost to individuals	Maximize	1: low costs (<100$); 2: moderate costs (<1000$); 3: high costs (>1000$)
Strategic and Operational Criteria (SOC)	SOC-01 –Capacity to detect and diagnose	Minimize	0: no tests, symptoms difficult to recognize; 1: distinct symptoms or existence of tests
	SOC-02 –Existence and effectiveness of current treatments	Minimize	0: no existing treatment; 1: partially effective treatment; 2: highly effective treatment available
	SOC-03 –Level of scientific knowledge of the disease	Minimize (diseases for which little is known of greater concern)	1: low–very little knowledge; 2: moderate–partial yet incomplete knowledge of disease symptoms, transmission, risk factors and treatment; 3: high–symptoms, transmission, risk factors and treatment well known
	SOC-04 –Optimization opportunities	Maximize	0: no opportunities; 1: potential opportunities
	SOC-05– Reportable disease	Maximize	0: not reportable; 1: nationally or internationally reportable

* Criteria added by stakeholders

### Criteria weighting

**Fig 2:**
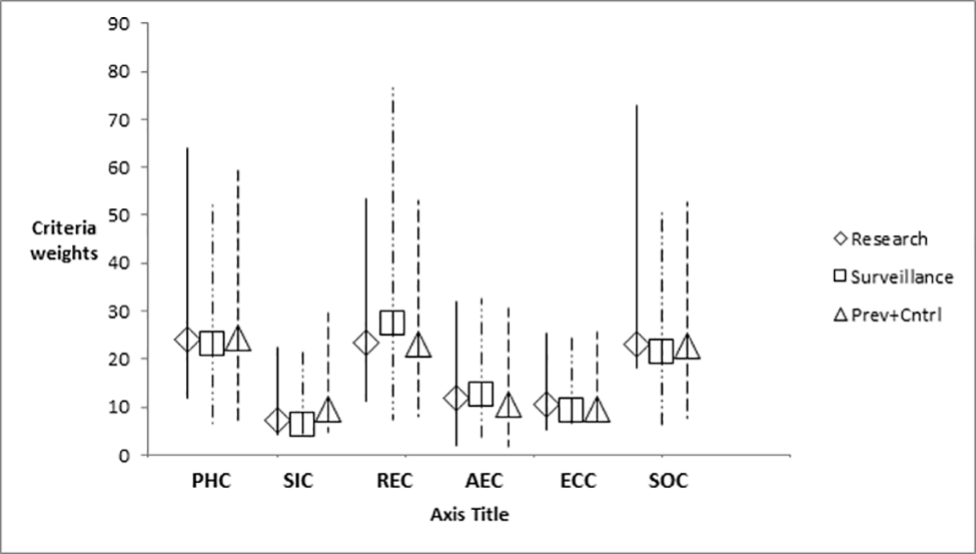
Criteria category weight average comparison by intervention domain. The span of stakeholder weights is indicated by the vertical lines with shaped makers indicating the intervention specific group means. Criteria categories are shown along the X axis with average weights by category shown along the Y axis. The differences between the weights given to each intervention domain (research, surveillance and prevention & control) were not found to be significantly different for any of the categories. Criteria category Legend (X axis): PHC: Public Health Criteria; SIC: Social Impact Criteria; REC: Risk and Epidemiology Criteria; AEC: Animal and Environmental Health Criteria; ECC: Economic Criteria; SOC: Strategic and Operational Criteria.

**Fig 3:**
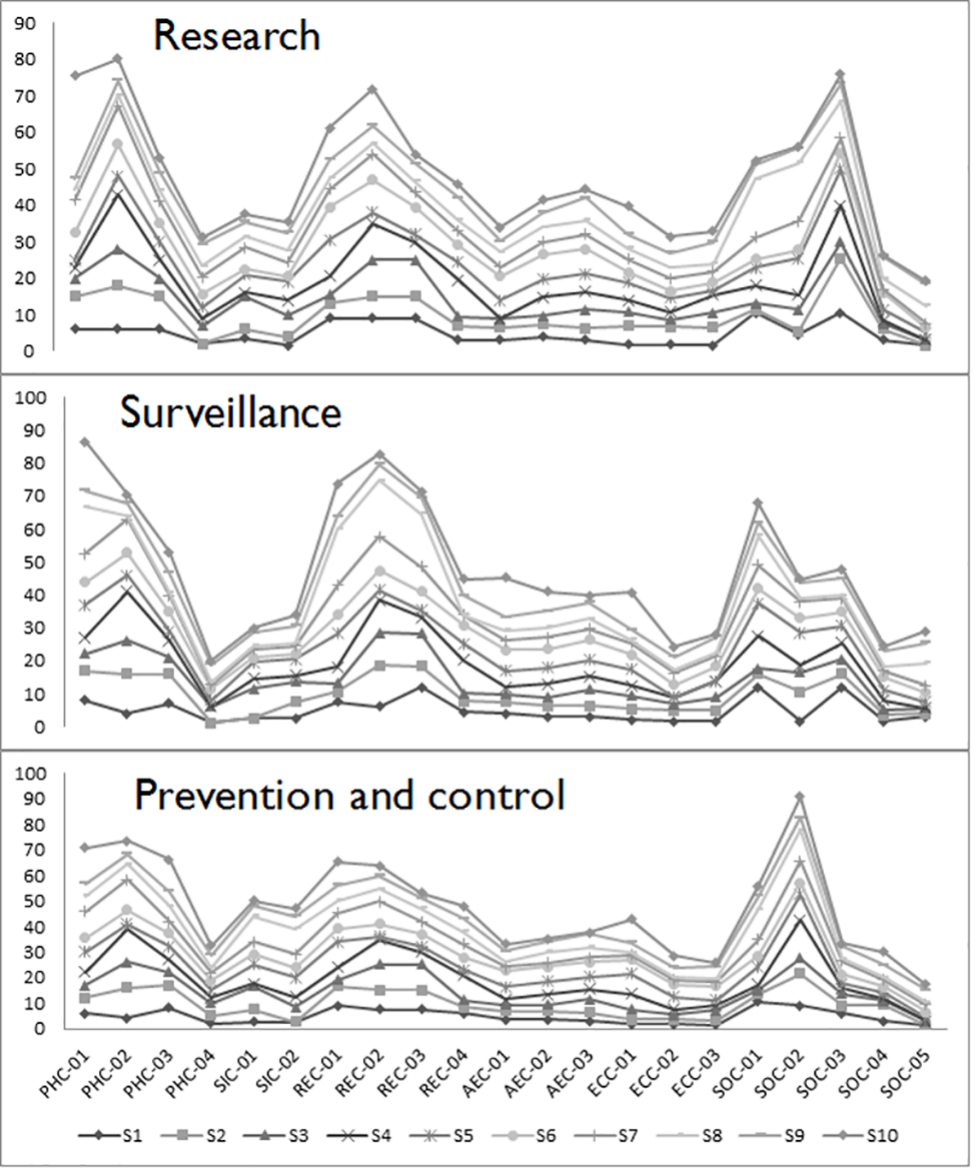
Individual weights by criteria and intervention domain. Each line in the graph represents each of the 10 Individual stakeholder’s (S1-S10) weight assignments on all 21 criteria. The relative importance of criteria within each category is seen to vary depending on the intervention domain. For example: the “SOC-03-level of knowledge” criterion received the most weight in the research domain, while the “SOC-01-capacity to detect disease” criterion received the most weight in the surveillance domain. The “SOC-02-Existence of treatment” received the most weight in the prevention and control domain.

**Fig 4:**
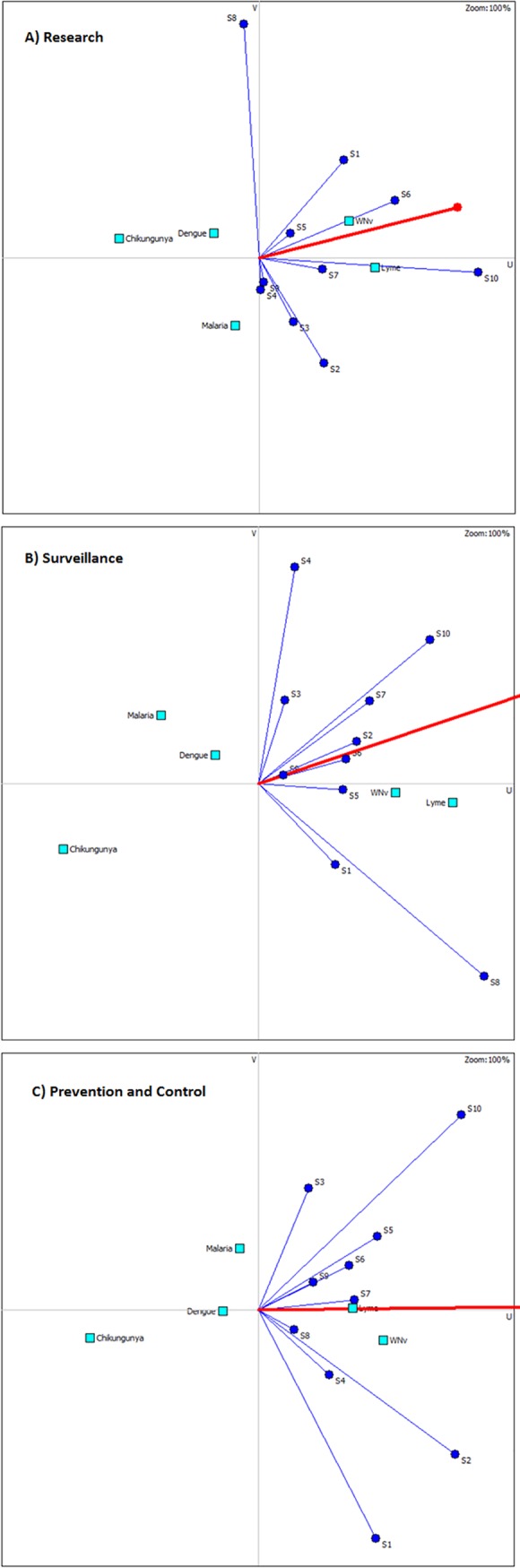
GAIA decision map for all intervention domains. Panel A) shows the GAIA map for the research domain, B) shows the surveillance domain and C) the prevention and control domain. In each map, the bold red line represents the group decision axis (i.e. consensus ranking) with the filled circle pointing in the direction of the group ranking. Square markers represent the ranking of the different diseases in *k*-dimensional space (where *k* represents the number of criteria) projected onto a 2-dimensional plane. Diseases closest to the group decision axis are prioritized over diseases further away from the decision axis. Stakeholders 1 through 10 are represented by the blue circular markers labelled S1-S10. Stakeholders pointing in the same direction as the group decision axis are most aligned with the group ranking. Stakeholders further away in space from each other and from the group decision axis have more disparate weighting tendencies and hence perspectives. For example, in panel A) S8 shows distinct weight position as compared with the rest of the stakeholders and therefore indicates a different set of values in this context as compared with the rest of the group. Clusters of stakeholders can also be observed occurring in each of the panels and indicate stakeholders with more similar weightings (i.e. perspectives). For example, in panel B) weights by stakeholders S2 and S6 are more similar to each other than to stakeholders S1 and S8.

Weighting of criteria was done individually by stakeholders and returned to the researchers by email in the weeks following the in-person focus group discussion. Ten of the original twelve stakeholders completed the criteria weighting exercise for each of the three intervention domains (research, surveillance or prevention and control). The relative importance of categories was generally similar for stakeholders across domains with no significant differences found between categories (Fig 2). Individual criteria weights were however found to differ within categories and across intervention domains (Fig 3). Stakeholder weights for all criteria are included in the supporting Information S2–S4 Tables. For all three intervention domains, the top three weighted categories were consistently “Public Health”, “Risk and Epidemiology” and “Strategic and Operational” criteria while the bottom three categories were consistently “Animal and Environmental Health”, “Economic” and “Social Impact” criteria. In the top 3, “Public Health” was generally the top weighted category in the subset with “Strategic and Operational” criteria consistently in 3^rd^ place whereas in the bottom 3, the “Animal and Environmental” criteria category was generally top rated while “Social Impact” was either last or tied for last. Despite similarities in the relative importance of categories, differences in individual weights were observed between stakeholders and are reflected in the GAIA visual analysis of projected stakeholder weights (Fig 4). The top weighted criterion varied considerably by individual and by intervention domain though was generally from one of the top 3 weighted categories (i.e. “Public Health”, “Strategic and Operational” or “Risk and Epidemiology” categories). The least weighted criterion also varied considerably by individual and intervention domain however, given the large number of criteria, multiple criteria often shared the lowest value but were not limited in origin to only the least weighted categories (“Animal and Environmental Health”, “Economic” and “Social Impact”). The weights expressed by stakeholders not directly associated with public health organisations (n = 3) were found to vary significantly (though not necessarily together) compared with the rest of the group. This was the case for the weights given to the “Public Health” category for research (p = 0.011) and surveillance (p = 0.016) interventions as well as the weights given to the “Risk and Epidemiology” category for prevention and control (p = 0.035) and research (p = 0.035) interventions. In the previously mentioned cases, the stakeholders not directly associated with public health generally attributed less weight to these categories compared to stakeholders directly working in public health. Conversely, the distribution of weights given to the “Social Impact” category for prevention and control interventions (p = 0.044) and for the “Strategic and Operational” category for research (p = 0.028) interventions were generally found to be higher for stakeholders not directly associated with public health.

The criterion “existence of favorable conditions for disease transmission” was weighted as the highest or second highest criterion within the “Risk and Epidemiology” category for the majority of stakeholders. However, across prioritization contexts, its relative importance varied depending on the stakeholder. This suggests that additional concerns (such as disease severity, level of scientific knowledge, current incidence and public risk perception) are given priority depending on the context and stakeholder. Criteria from the “Social Impact”, “Animal and Environmental Health” as well as “Economic” categories were rarely among the top 5 weighted individual criterion for stakeholders, though these do appear among the top 5 for some stakeholders across each of the intervention domains. The small number of stakeholders and difference in perspective between groups of stakeholders is likely driving this observation.

### Pilot prioritization of diseases

From the pilot prioritization exercise, *West Nile virus and Lyme* were ranked 1^st^ and 2^nd^ respectively in the research and prevention and control domains, with this order reversed in the surveillance domain. *Dengue*, *Malaria*, *and Chikungunya virus* were ranked 3^rd^, 4^th^ and 5^th^ in the research and surveillance domains with *Malaria* in 3^rd^ and *Dengue* in 4^th^ in prevention and control domain. Diseases were ranked with and without stakeholder assigned weights across intervention domains to assess dominance. No single disease was found to be dominant. Varying the disease evaluations showed sensitivity of disease rankings to their evaluations. Furthermore, different scales would produce different evaluations with potentially different rankings. As the goal of the current project was not to formally assess local disease priorities (for the creation of an official list of priority diseases), but rather to assess differences in stakeholder perspectives and examine the effect of these on potential rankings, a formal systematic assessment of sensitivity to disease scales was not performed. The retained scales allowed us to distinguish between the relative importance of diseases per criterion. Unweighted uni-criterion analysis revealed criteria for which each of the assessed diseases ranked 1st at least once. For instance, *Dengue* ranked 1st on the “capacity to detect and diagnose” criterion, *Chikungunya* ranked 1st on the “level of scientific knowledge of the disease” criterion, *Lyme* ranked 1st on the “current global trend” criterion, *malaria* ranked 1st on the “risk perception of the public” criterion and *West Nile virus* ranked 1st on the “epidemic potential” criterion. Group and individual stakeholder ranking results are shown in Table 4 with corresponding assessment values used (based on context specific data obtained from the literature) shown in the evaluation matrix in Table 5 (supporting references used for disease assessments are provided in supporting information S1 Appendix).

**Table 4:** Pilot prioritization of diseases for the group and by stakeholder for each intervention domain.

	GRP		S1		S2		S3		S4		S5		S6		S7		S8		S9		S10	
Diseases	Rnk	Phi	Rnk	Phi	Rnk	Phi	Rnk	Phi	Rnk	Phi	Rnk	Phi	Rnk	Phi	Rnk	Phi	Rnk	Phi	Rnk	Phi	Rnk	Phi
**Research**																						
West Nile virus (WNV)	1	0.08	2	0.09	3	-0.01	3	0.02	3	-0.00	1	0.10	2	0.17	1	0.10	1	0.09	4	-0.01	2	0.31
Lyme (LYM)	2	0.07	1	0.13	1	0.14	2	0.04	4	-0.03	3	-0.01	1	0.18	2	0.03	4	-0.06	3	0.00	1	0.23
Dengue (DEN)	3	-0.01	3	-0.02	4	-0.04	4	-0.03	2	0.04	4	-0.03	3	-0.07	4	0.01	2	0.08	2	0.01	4	-0.15
Malaria (MAL)	4	-0.02	4	-0.12	2	0.05	1	0.05	1	0.05	2	0.00	4	-0.08	3	0.02	5	-0.18	1	0.02	3	-0.05
Chikungunya (CHIKV)	5	-0.11	5	-0.13	5	-0.13	5	-0.08	5	-0.06	5	-0.06	5	-0.20	5	-0.16	3	0.07	5	-0.03	5	-0.35
**Surveillance**																						
West Nile virus (WNV)	2	0.10	2	0.02	1	0.15	3	0.02	4	0.00	1	0.15	2	0.10	2	0.15	2	0.26	1	0.04	2	0.16
Lyme (LYM)	1	0.14	1	0.18	2	0.10	2	0.04	3	0.03	2	0.08	1	0.13	1	0.13	1	0.38	2	0.03	1	0.27
Dengue (DEN)	3	-0.02	3	0.00	3	-0.01	4	-0.03	1	0.08	3	-0.01	3	-0.02	3	-0.00	3	-0.13	3	-0.01	4	-0.07
Malaria (MAL)	4	-0.06	5	-0.12	4	-0.07	1	0.05	2	0.06	4	-0.09	4	-0.06	4	-0.05	5	-0.27	4	-0.01	3	-0.03
Chikungunya (CHIKV)	5	-0.16	4	-0.08	5	-0.18	5	-0.08	5	-0.18	5	-0.13	5	-0.15	5	-0.22	4	-0.25	5	-0.04	5	-0.33
**Prevention & control**																						
West Nile virus (WNV)	1	0.10	1	0.14	1	0.21	3	0.02	1	0.12	1	0.10	2	0.05	1	0.10	2	0.05	1	0.05	2	0.15
Lyme (LYM)	2	0.06	2	0.11	2	0.15	2	0.04	3	-0.02	2	0.07	1	0.09	2	0.03	4	-0.03	2	0.04	1	0.14
Dengue (DEN)	4	-0.02	3	-0.03	4	-0.09	4	-0.03	2	0.02	4	-0.02	3	-0.00	3	0.02	1	0.06	4	-0.02	4	-0.07
Malaria (MAL)	3	-0.01	5	-0.12	3	-0.08	1	0.05	4	-0.03	3	0.02	4	-0.01	4	-0.01	3	-0.02	3	0.01	3	0.06
Chikungunya (CHIKV)	5	-0.13	4	-0.10	5	-0.19	5	-0.08	5	-0.09	5	-0.16	5	-0.13	5	-0.14	5	-0.07	5	-0.07	5	-0.28

GRP–overall group ranking; Rnk–rank; S1-S10 –denotes stakeholders 1 through 10; Phi–net outranking flows (combined positive and negative flows) indicating performance of each disease

**Table 5:** Disease evaluation matrix.

Diseases	Criteria																				
	PHC1	PHC2	PHC3	PHC4	SIC1	SIC2	REC1	REC2	REC3	REC4	AEC1	AEC2	AEC3	ECC1	ECC2	ECC3	SOC1	SOC2	SOC3	SOC4	SOC5
West Nile virus (WNv)	2	2	1	1	1	2	3	2	1	5	6	4	2	1	1	1	1	0	3	1	1
Lyme (LYM)	3	2	1	1	1	2	3	1	3	5	6	2	2	2	1	2	1	1	3	1	1
Dengue (DENV)	0	4	0	1	1	1	1	1	2	2	0	1	2	3	2	1	0	1	3	1	1
Malaria (MAL)	0	4	1	2	2	1	2	1	1	2	0	0	2	3	3	1	1	2	3	1	1
Chikungunya (CHIKV)	0	1	0	1	1	1	1	1	2	2	0	1	2	1	1	2	1	0	2	1	1

Disease evaluation matrix showing evaluation scores for each of the five pilot diseases based on context specific data reviewed pertaining to each disease over all criteria. Note: Criteria AEC3, SOC4 and SOC5 are non-discriminating with the above data set due to lack of variation between disease evaluation values but could be discriminating with different diseases or more refined data set. Criteria were retained in the model due to expressed interest of stakeholders. PHC–Public health criteria; SIC–Social impact criteria; REC–Risk and epidemiology criteria; AEC–Animal and environmental health criteria; ECC–Economic criteria; SOC–Strategic and operational criteria

Sensitivity analysis results with weight stability intervals for all criteria by all stakeholders for the research domain are shown in Table 6 in descending order of stability (from least stable to most stable). Five of the twenty-one criteria were found to be very stable as per the size of their stability intervals spanning almost the entire range of possible values from 0–100 for all stakeholders for the 1^st^ order ranking. This indicates that the rank ordering of diseases would not change for any weight value given to these criteria between 0 and 100. These five criteria were “the existence of a vulnerable group”, “potential to increase social inequality”, “ability to infect the environment”, “optimization opportunities” and “reportable disease”. The remaining criteria were found to have relatively small stability intervals (<10 points) for at least one stakeholder indicating high sensitivity to assigned weights by stakeholders. The “current trend”, “cost to individuals” and “general knowledge” criteria were found to be the highly sensitive for 7, 6 and 5 stakeholders respectively. This was closely followed by “existence of favourable conditions”, “disease severity for animals”, “cost to private sector” and “public risk perception” criteria for at least 4 stakeholders. Surveillance and prevention and control sensitivity analysis results were similar and are included in the supplementary material S5 and S6 Tables.

**Table 6:** Weight stability intervals in descending order from sensitivity analysis of all stakeholders for the research domain.

Criteria	S1	S2	S3	S4	S5	S6	S7	S8	S9	S10
REC-03	9 (0–10)	6(2–100)	10 (0–11)	5 (0–5.5)	2 (0–8)	4 (0–10)	5 (2–6)	3 (0–4)	5 (0–6)	3 (0–6)
ECC-03	1 (0–2.5)	5 (0–100)	4 (0–6)	5 (0–11)	1 (0–9)	3 (0–11)	2 (0–100)	2 (0–4)	6 (0–8)	3 (0–8)
SOC-02	4 (3–100)	1 (0–10)	6 (0–7)	4 (0–5)	10 (4–100)	8 (2.5–100)	9 (6–11)	16 (15–100)	4 (0–5)	1 (0–100)
REC-01	9 (0–100)	4 (0–100)	3 (0–4.5)	5 (4–10)	10 (1.5–100)	5 (0–100)	6 (4.5–11)	3 (2.5–100)	5 (3.5–8)	9 (0–100)
AEC-02	4 (1.5–100)	3(0–20)	3 (0–3)	5 (0–5.5)	5 (0–100)	4 (0–100)	2 (0–4)	4 (3–100)	4 (0–5)	4 (0–100)
ECC-02	2 (0–14)	5(0–9)	2 (0.5–100)	2 (1–100)	4 (0–10)	4 (0–9)	2 (0–100)	3 (0–4)	4 (2.5–100)	4 (0–23)
SOC-01	10 (0–20)	1 (0–12)	1 (0–8)	5 (0–5.5)	5 (0–14)	6 (0–13)	6 (0–7)	16 (0–17)	4 (0–5)	1 (0–27)
PHC-01	6 (0–100)	9 (4–100)	5 (0–6)	3 (0–6)	2 (0–100)	9 (1–100)	11 (0–100)	3 (2–100)	3 (0–5)	28 (0–100)
ECC-01	2 (0–3)	5(0–13)	4 (2–100)	3 (0–100)	5 (0–11)	4 (0–9)	2 (0–100)	3 (0–3.5)	4 (2.5–100)	7 (0–15)
SIC-02	1 (0–12)	3 (0–7)	6 (4.5–100)	4 (0.5–100)	5 (0–12)	4 (0–11)	5 (0–16)	3 (0–4)	5 (3–100)	3 (0–25)
REC-04	3 (0–13)	4 (0–9)	3 (1–100)	10 (6–100)	5 (0–12)	4 (0–11)	5 (0–100)	3 (0–4)	6 (4–100)	4 (0–60)
AEC-01	3 (0–100)	3(0–100)	3 (0–4)	0 (0–100)	5 (0–100)	3 (0–100)	2 (0–100)	4 (3–100)	3 (0–5)	4 (0–100)
PHC-02	6 (0–18)	12 (0–17)	10 (8.5–100)	15 (11–100)	5 (0–14)	10 (0–18)	12 (3.5–16)	3 (0–4)	4 (1.5–100)	6 (0–31)
SIC-01	3 (0–19)	3 (0–7)	9 (7–100)	1 (0.5–100)	5 (0–12)	6 (0–13)	5 (3–16)	3 (0–20.5)	4 (3–100)	2 (0–24)
REC-02	9 (8–100)	6 (0–13)	10 (0–11)	10 (0–13)	3 (0–100)	7 (0–100)	9 (0–12)	3 (2.5–100)	5 (0–7)	10 (5–100)
SOC-03	10 (0–25)	15 (0–29)	4 (0–13)	10 (0–17)	10 (0–20)	4 (0–21)	5 (1–13.5)	10 (0–12)	5 (0–9)	3 (0–36)
PHC-03	6 (0–100)	9 (0–100)	5 (0–100)	5 (4–100)	5 (0–100)	6 (0–100)	5 (3–100)	3 (2–100)	5 (4–100)	4 (0–100)
PHC-04	2 (1–100)	0 (0–100)	5 (3–100)	2 (0–100)	3 (0–100)	4 (0–100)	3 (0–100)	3 (0–100)	6 (3.5–100)	2 (0–100)
AEC-03	3 (0–100)	3(0–100)	5 (0–100)	5 (4.5–100)	5 (0–100)	4 (0–100)	2 (0–100)	4 (0–100)	6 (0–100)	3 (0–100)
SOC-04	3 (0–100)	3 (0–100)	1 (0–100)	1 (0–100)	3 (0–100)	4 (0–100)	1 (0–100)	3 (0–100)	6 (0–100)	0 (0–100)
SOC-05	1 (0–100)	0 (0–100)	1 (0–100)	0 (0–100)	2 (0–100)	1 (0–100)	1 (0–100)	5 (0–100)	6 (0–100)	1 (0–100)

S1-S10 –denotes stakeholders 1 through 10; Stakeholder assigned weights are given for all criteria followed by the stability interval in parentheses over which the ranking order for the 1^st^ position items are maintained. PHC–Public Health criteria; SIC–Social impact criteria; REC–Risk and epidemiology criteria; AEC–Animal and environmental health criteria; ECC—Economic criteria; SOC–Strategic and operational criteria

## Discussion

The current study solicited Quebec stakeholder perspectives and concerns for vector-borne diseases management under climate change. These concerns were translated into criteria and used to construct prioritization models to rank vector-borne diseases in the context of public health interventions pertaining to research, surveillance and, prevention and control. The prioritization models consisted of 21 criteria and were accompanied by corresponding stakeholder weights that varied depending on the intervention context (research, surveillance or prevention and control). Criteria were distributed across six categories: “public health”, “risk and epidemiology”, “strategic and operational”, “animal and environmental health”, “economic” and “social impact” with the relative importance of criteria within these categories varying depending on the intervention context. A pilot prioritization exercise showed how resulting disease priorities also change depending on the intervention context.

What have we learned from years of prioritization exercises? The use of an initial literature review followed by a stakeholder consultation in our study allowed for the inclusion of a broad range of considerations which were factored into our prioritization models. Our literature review suggests that common criteria and categories recur across studies [7–21]. This may be due to shared learning from previously published studies, but may also be representative of core concerns that are shared across scales and regions which translate into a set of common decision criteria that remain applicable across public health contexts and that can be used as a basis for prioritization models as we have done here. Furthermore, these models were found to be equally applicable in three intervention contexts with associated stakeholder specific weights. While our study included similar categories of criteria to previous prioritization exercises, detailed direct comparisons cannot be made between studies since the prioritization objectives and approaches differed.

What is the value of including stakeholders with a variety of backgrounds in the process? Few studies involve the public in their consultation process, and generally involve only a narrow range of participants in the process. Involvement of a diversity of participants (researchers, government, public health personnel, and non-technical citizens) in the criteria and preference elicitation process can help ensure that a broad set of value perspectives are considered. An attempt at including a broader range of voices in the vector-borne disease prioritization process has been done in the current study. While previous studies have contrasted public and expert rankings [28,29], the current study demonstrated a potential method for how to include these voices in the same consultation exercise in order to provide an opportunity for shared knowledge exchange and discussion of concerns between groups. Including multiple stakeholders with diverse backgrounds helps broaden the perspectives considered and contributes to the inclusion of a representative set of societal concerns and perspectives in the decision analysis process. The weighting process also allows a second measure of where stakeholders stand with regards to these concerns. Weights by non public health related organisations were found to be significantly different from the rest of the group and may in part be explained by differences in perceived responsibility or accountability between these groups [50]. Where differences occur between groups may indicate points of entry for future research into how differences in perspective impact intervention acceptance or uptake down the line. While public health officials are a part of society, their knowledge may push them to dismiss a disease which causes great alarm to the general public (e.g. one with high severity but low risk of transmission in the current context). Understanding what is being factored into each group’s decision making equation is therefore useful in order to inform future interventions and public health actions.

### Remaining challenges

From a public policy point of view, ensuring that resulting decisions are well aligned with publicly held values should be of interest and is something that is possible to explore with the current approach. The use of formal prioritization approaches is evolving. Who to include in the process is a consideration that should be regularly revisited by decision makers. The challenge becomes how to process the range of viewpoints consulted during analysis. Should different viewpoints be weighted equally? Should all groups be consulted primarily to elicit the most comprehensive range of viewpoints or are related weightings by different stakeholder groups useful and necessary? These questions should be addressed early on in the prioritization exercise and responses are likely to vary depending on the specific prioritization context. Key participant profiles representing the viewpoints of various societal groups can be constructed and has been used before in environmental decision making processes [51,52]. The consistency of weighting tendencies within stakeholder groups was not assessed in the current study given the small number of participants; however, given a larger sampling, the consistency of weighting trends within groups could be further examined.

This work is still in the early stages of understanding the impact of including broader consultation on these issues and requires further exploration to help guide the minimal set of consultations that should be considered. For certain intervention domains, such as surveillance, public health perspectives may be one of the most important to take into account; however, with regards to publicly funded research interventions, it may be of interest to ensure that resulting decision priorities are coherent with socially held values. While public health officials themselves do represent socially held values, their unique perspective may differ from that of society at large and effort should be made to examine where potential differences may exist. Stakeholders outside of public health, especially non-government stakeholders, offer more than just another view and opinion in this process. They help take into account socially held values, but also ensure that interventions are feasible in the field. Their inclusion is a first step in the communication and knowledge transfer process with these organizations, such as municipalities. With regards to the disease prevention and control domain, understanding and attempting to integrate different perspectives may help pinpoint areas where additional information needs exist. For example, diseases which may pose significant health and economic impacts to livestock populations but few to human populations may fall under the radar of human public health officials. Including stakeholders from other domains such as animal health and environmental health may help to ensure that attention is given to such cases. In this exercise, significant differences were found for the “social impact” and “risk and epidemiology” categories suggesting that important differences may exist between public health experts and the general public with regards to risk perception, knowledge and risk assessment of disease threats. Differences in risk perception between the general public and experts have been observed in previous studies [53–55].

### Limitations

This study did not aim to predict which diseases might be sensitive to climate change, but rather set out to identify the primary concerns (i.e. criteria) of a cross-section of society with regards to vector-borne disease management in these contexts. Here “cross section of society” refers not to the number of stakeholders engaged, but rather the diversity of organizations consulted. Furthermore, the pilot prioritization presented was for illustration purposes only and should not be interpreted as a formal assessment of local Quebec priorities. Additional data as well as further discussion with experts and stakeholders is required to validate these findings.

With regards to stakeholders included in this study, these groups were invited based on their representation of a cross-section of society at large and meant to represent perspectives from various interest groups beyond that of public health alone (e.g. patient rights group, senior citizens). Of the 24 invited, only 12 were able to participate due to availability. Of those 12, only 10 further completed the weighting exercise, once again due to time constraints. A larger number of participants with different backgrounds or organizational membership would likely have increased the diversity of perspectives included both in terms of criteria considered and weightings assigned to those criteria. Furthermore, while this same group of stakeholders also participated in a concurrent study on West Nile virus, additional evaluations with different stakeholder groups will be needed to assess whether weightings were strongly influenced by this factor. It should be noted that disease rankings are not driven by stakeholder weightings alone, but rather the combined evaluation of literature evidence with stakeholder weights. The relative stability of the rankings was explored in sensitivity analyses presented the results section (Table 6 and supplemental materials S5 and S6 Tables) and shown to be relatively stable for many of the criteria in the final models.

### Future prioritization exercises

The current study offers preliminary models that can be used as a starting point for future prioritization exercises to build on. Thorough reflection should be held on the purpose or objective of the prioritization exercise and who the most representative stakeholders are to include in that context. The approach used in the current study offers an opportunity to identify concerns held by participating stakeholders and provides a method to examine the effect of these differences on disease rankings. The inclusion of diverse perspectives provides opportunities to identify knowledge gaps and data needs that go beyond an assessment of available scientific evidence alone and thus contributes to a more comprehensive process informing decision. In allocating health resources, a formal process for evaluating diverse evidence and interests notably contributes to enhancing and articulating the rigour, transparency, and acceptability of the decision process, in a manner which is compatible with currently held social values. In other words, what, in practice, decision making will gain from this approach includes: 1) a more comprehensive view of the issue–issues that decision makers did not think existed or did not think were important; 2) a sense of perceived risk (above and beyond what the usual experts / specialists are saying); 3) a level of acceptability by using standard criteria to prioritize health risks; 4) a sense of transparency as to how the prioritization results were obtained; 5) a level of rigour to the results given that the prioritization follows a set of transparent and accepted rules. The approach described here is coherent with the vulnerability and adaptation framework described in the 4^th^ IPCC assessment report [56] and more specifically, the impact assessment stage in terms of providing a mechanism with which to narrow a list of diseases to focus on in the context of climate change.

## Supporting information

S1 Table Criteria trace summary. (DOCX)

S2 Table Individual stakeholder weights for all criteria ordered by importance for the research intervention domain. (DOCX)

S3 Table Individual stakeholder weights for all criteria ordered by importance for the surveillance intervention domain. (DOCX)

S4 Table Individual stakeholder weights for all criteria ordered by importance for the prevention and control intervention domain. (DOCX)

S5 Table Weight stability interval sensitivity analysis for all stakeholders for the surveillance domain. (DOCX)

S6 Table Weight stability interval sensitivity analysis for all stakeholders for the prevention & control domain. (DOCX)

S1 Appendix Supporting references used to assess disease scores for the pilot prioritization. (DOCX)
