# Supplementary material for: Criteria for the prioritization of public health interventions for climate-sensitive vector-borne diseases in Quebec
Source: PLoS One. 2017 Dec 27;12(12):e0190049. doi: 10.1371/journal.pone.0190049 (PMC5744945; doi:10.1371/journal.pone.0190049)
Supplement: S1 Table — (DOCX) [file pone.0190049.s001.docx]

# S1 Table. Criteria Trace Summary

| Cat | # | Criteria |  |  |  |  |  |  |  |  |  |  |  |  |  |  |  |  |  |  |  |  |  |  |  |  |  | New criteria |
| --- | --- | --- | --- | --- | --- | --- | --- | --- | --- | --- | --- | --- | --- | --- | --- | --- | --- | --- | --- | --- | --- | --- | --- | --- | --- | --- | --- | --- |
|  |  |  | *Carter 1991 | *Rushdy et al 1998 | Weinberg et al 1999 | Doherty 2000 | Horby et al 2001 | *Valenciano 2002 | WHO 2003 (Dubrovnik pledge) | WHO 2006 | Doherty 2006 | McKenzie et al 2007 | *Krause et al 2008 (a&b) | Cardoen et al 2009 | Capek 2010 | Havelaar et al 2010 | Pavlin et al 2010 | Ruzante et al 2010 | Balabanova et al 2011 | Humblet et al 2012 | Ng & Sargeant 2012, 2013 | Ng & Sargeant 2013 | Cediel et al 2013 | Cox et al 2013 | Del rio Vilas 2013 | Brookes et al 2014 | Kadohira 2015 |  |
| PH | 21 | Incidence or prevalence, occurrence in humans, burden of ill health, illness rate, % | 1 | 1 | - | 1 | 1 |  | 1 | 1 | 1 | - | 1 | 1 | 1 | - | 1 | 1 | 1 | 2 | 2 | 1 | 1 | 1 | - | 1 | 1 | PHC-01 |
| PH | 19 | Morbidity, severity of symptoms, consequences for humans, work and school absenteeism, impact on quality of life, Clinical course | 1 | - | - | 1 | - |  | - | - | 1 | 1 | 1 | 1 | 1 | 1 | 1 | 1 | 1 | 2 | 1 | 1 | 1 | 1 | 1 | 1 | - | PHC-02 |
| PH | 4 | Duration of illness, chronicity of illness or sequelae | - | - |  | - | - |  | - | - | - | - | - | - | - | - | - | - | 1 | 1 | 1 | 1 | - | - | - | - | - | PHC-02 |
| PH | 14 | Deaths, mortality, death-case ratio, case fatality rate (humans), fatality | 2 | - | - | - | - |  | - | 1 | - | - | 1 | - | - | 1 | 1 | 1 | 1 | 2 | 1 | 1 | 1 | 1 | - | 1 | 1 | PHC-02 |
| SP | 16.5 | Public concern & perception of risk, social sensitivity, public attention | 1 | 1 | - | 1 | 1 |  | 1 | 1 | 1 | - | 0.5 | - | - | - | 0.5 | 2 | 1 | 2 | 2 | - | 0.5 | - | 1 | - | - | SIC-01 |
| SP | 1 | Public awareness | - | - | - | - | - |  | - | - | - | - | - | - | - | - | - | - | - | - | 1 | - | - |  |  |  | - | SIC-02 |
| LO | 2 | Potential to drive public health policy, political impact of disease in humans | - | - | - | 1 | - |  | - | - | - | - | - | - | - | - | - | - | - | 0 | 1 |  | - |  |  | - | - |  |
| RE | 9.5 | Communicability, potential to spread to general population, human-to-human spread | 1 | - | - | 1 | - |  | - | - | 1 | 1 | - | - | 0.5 | 1 | - | - | - | 0 | 2 | 1 | - |  |  | - | 1 | REC-02 |
| RE | 8 | Epidemic or potential for outbreaks | 1 | - | - | - | - |  | - | 1 | 1 | - | 1 | - | 1 | - | 1 | - | - | 1 | - | - | 1 | - | - | - | - | REC-02 |
| RE | 8.5 | Appearing to change over time, trend | - | - | - | 1 | - |  | - | - | 1 | - | 1 | - | - | - | 0.5 | - | 1 | 0 | 1 | 1 | 1 | 1 | - | - | - | REC-02 |
| RE | 10.5 | Emerging potential, prob. of introduction | - | 1 | 1 | - | 1 |  | 1 | 1 | - | 1 | 1 | - | - | 1 | 0.5 | - | - | 0 | - | - | 1 |  |  | - | 1 | REC-01 |
| RE | 7 | Mode of transmission, speed of spread | - | - | - | - | - |  | - | - | - | - | - | - | 1 | - | - | - | - | 3 | 3 |  | - |  |  | - | - |  |
| AE | 11 | Occurrence in animals | - | - | - | - | - |  | - | - | - | 4 | - | 1 | 1 | 1 | - | - | - | 7 | 3 | 1 | - | 2 | - | - | - | AEC-01 |
| AE | 4.5 | Consequences of spread in animals, severity | - | - | - | - | - |  | - | - | - | 4 | - | 0.5 | - | - | - | - | - | 0 | 1 | 1 | - | 1 | - | - | - | AEC-02 |
| AE | 3 | Animal Case fatality rate (mortality) |  |  |  |  |  |  |  |  |  |  | - | - | - | - |  |  | - | - |  | 1 | - | 1 | - | 1 | - | AEC-02 |
| EC | 16.5 | Socioeconomic impact, market loss, econ. damage | 1 | 1 | - | 1 | 1 |  | 1 | 1 | 1 | - | - | 0.5 | 1 | 1 | - | 1 | - | 6 | 3 | 2 | - | 1 |  | 2 | - |  |
| EC | 2 | cost of illness | - | - | - | - | - |  | - | - | - | - | - | - | - | - | - | 1 | - | 2 | - | - | - |  |  | - | - |  |
| EC | 1 | Govt compensation to industry (to compensate for losses) | - | - | - | - | - |  | - | - | - | - | - | - | - | - | - | - | - | 0 | - | - | - | - | - | 1 | - |  |
| EC | 1 | Econ Impact on animal industry |  |  |  |  |  |  |  |  |  |  |  |  |  |  |  |  | - | 7 |  |  |  |  |  |  | - |  |
| LO | 15.5 | Preventability, health gain opportunity | 1 | 1 | - | 1 | 1 |  | 1 | 1 | 1 | - | 1 | - | 2 | - | 1 | - | 1 | 3 | 0.5 | - | 1 | 1 | - | - | 1 |  |
| LO | 9.5 | Treatability, treatment possibilities and needs (in humans) (including AMR) | - | - | - | - | - |  | - | - | - | - | 1 | - | 1 | - | 1 | - | 1 | 2 | 0.5 | - | 1 | 1 | - | - | 1 |  |
| LO | 6 | Diagnostic ability (and quality) in humans |  |  |  |  |  |  |  |  |  |  |  |  |  | - |  |  |  | 2 | 3 |  |  | 1 | - |  | 1 |  |
| LO | 7.5 | International considerations, notification regulations in national public health, duties, obligations | 1 | - | - | 1 | - |  | - | 1 | 1 | - | 0.5 | - | 2 | - | 0.5 | - | - | 0 | - |  | 0.5 | - | - | - | - |  |
| LO | 4 | Scientific Knowledge of pathogenic agent |  |  |  |  |  |  |  |  |  |  |  |  |  |  |  |  |  | 2 | 1 | 1 |  | 1 |  |  | - |  |
| LO | 4 | Immediate public health response necessary, timeliness | 1 | - | - | - | - |  | 1 | - | 1 | - | - | - | - | - | 1 | - | - | 0 | - |  | - |  |  | - | - |  |
| LO | 2 | Other sector interest (incl. Agriculture Canada ) | 1 | - | - | 1 | - |  | - | - | - | - | - | - | - | - | - | - | - | 0 | - |  | - |  |  | - | - |  |
| RE | 4 | Evidence for pathogenesis | - | - | - | - | - |  | - | - | - | - | 1 | - | - | - | - | - | - | 0 | 1 |  | 1 | 1 | - | - | - | REC-02 |
| RE | 2 | Current geographical distribution in region of interest and risk of expansion |  |  | - |  |  |  |  |  |  |  | - | - | 1 | - |  |  | - | 0 | 1 |  | - | 1 | - | - | - | REC-02 |
| PH | 5.5 | Evidence for risk factors, high risk groups |  |  | - |  |  |  |  |  |  |  | 1 | - | 0.5 | - | 1 | - | - | 0 | 1 | 1 | 1 | - | - | - | - |  |
| PH | 2 | Health care utilisation, proportion of events requiring public health action | - | - | - | - | - |  | - | - | - | - | - | - | - | - | - | - | 2 | 1 | - |  | - | - | - | - | - | PHC-02 |
| LO | 2 | Elimination potential (in humans) | - | - | - | - | - |  | - | - | - | - | - | - | - | - | 1 | - | - | 0 | 1 |  | - |  |  | - | - |  |
| LO | 2 | Validity of epidemiological information | - | - | - | - | - |  | - | - | - | - | 1 | - | - | - | - | - | - | 0 | - |  | 1 | - | - | - | - |  |
| LO | 2 | Existence of surveillance or studies in animals or vectors | - | - | - | - | - |  | - | - | - | - | - | - | 2 | - | - | - | - | 0 | 1 |  | - | - | - | - | - |  |
| RE | 3 | Transmission potential between animals |  |  |  |  |  |  |  |  |  |  |  |  |  |  |  |  | - | 1 | 1 | 1 |  |  |  |  |  |  |
| RE | 4 | Transmission potential from Animal-human, zoonotic potential | - | - |  | - | - |  | - | - | - | - | - | - | - | 1 | - | - | - | 0 | 1 | 1 | - | 1 |  | - | - | REC-02 |
| LO | 2 | Treatment possibilities in animals |  |  |  |  |  |  |  |  |  |  |  |  |  | - |  |  |  | - | 1 |  |  | 1 | - |  | - |  |
| AE | 2 | High-risk groups in animals |  |  |  |  |  |  |  |  |  |  |  |  |  |  |  |  | - |  | 1 | 1 |  |  |  |  |  |  |
| AE | 2 | impact on animal welfare and biodiversity |  |  |  |  |  |  |  |  |  |  | - | - | - | - |  |  | - | 1 |  |  | - | - | 1 | - | - | AEC-02 |
| RE | 2 | Transmission potential from humans to animals |  |  |  |  |  |  |  |  |  |  |  |  |  |  |  |  |  |  | 1 | 1 |  |  |  |  |  | REC-02 |
| RE | 2 | Animal Trend last 5 years |  |  |  |  |  |  |  |  |  |  |  |  |  | - |  |  | - | - |  | 1 |  | 1 | - |  | - | REC-02 |
| AE | 1 | Specific animals |  |  |  |  |  |  |  |  |  |  |  |  |  |  |  |  | - |  | 1 |  |  |  |  |  |  |  |
| AE | 1 | Animal attack rate |  |  |  |  |  |  |  |  |  |  | - | - | - | - |  |  | - | - |  |  | - | - | - | 1 | - | AEC-02 |
| AE | 1 | Classification of zoonoses |  |  |  |  |  |  |  |  |  |  |  |  |  |  |  |  | - | 1 |  |  |  |  |  |  | - |  |
| AE | 1 | Lower human consumption of animals |  |  |  |  |  |  |  |  |  |  |  |  |  |  |  |  | - | 1 |  |  |  |  |  |  | - |  |
| AE | 1 | Zoonotic / common agent |  |  |  |  |  |  |  |  |  |  |  |  |  |  |  |  | - | 1 |  |  |  |  |  |  | - |  |
| AE | 1 | Likely incidence in domestic animals |  |  |  |  |  |  |  |  |  |  |  |  |  | - |  |  | - | - |  |  |  | 1 | - |  | - | AEC-01 |
| AE | 1 | Pathogenicity in domestic animals |  |  |  |  |  |  |  |  |  |  |  |  |  | - |  |  | - | - |  |  |  | 1 | - |  | - |  |
| AE | 1 | Potential environmental impact |  |  |  |  |  |  |  |  |  |  |  |  |  | - |  |  | - | - |  |  |  | 1 | - |  | - |  |
| AE | 1 | Potential social impact |  |  |  |  |  |  |  |  |  |  |  |  |  | - |  |  | - | - |  |  |  | 1 | - |  | - |  |
| EC | 1 | Impact on int’l trade |  |  |  |  |  |  |  |  |  |  |  |  |  | - |  |  | - | - |  |  |  |  | 1 | - | - |  |
| LO | 1 | Simplicity, sustainability | - | - | - | - | - |  | - | - | - | - | - | - | - | - | 1 | - | - | 0 | - |  | - |  |  | - |  |  |
| LO | 1 | Existing control measures, or surveillance programs | - | - | - | - | - |  | - | - | - | - | - | - | 1 | - | - | - | - | 0 |  |  | - | - | - | - | - |  |
| LO | 1 | Previous classification status | - | - | - | - | - |  | - | - | - | - | - | - | 1 | - | - | - | - | 0 |  |  | - | - | - | - | - |  |
| LO | 1 | Surveillance feasibility | - | - | - | - | - |  | - | - | - | - | - | - | 1 | - | - | - | - | 0 |  |  | - | - | - | - | - |  |
| LO | 1 | Diagnostic ability (and quality) in animals |  |  |  |  |  |  |  |  |  |  |  |  |  |  |  |  |  |  | 1 |  |  |  |  |  |  |  |
| LO | 1 | Control measures animals |  |  |  |  |  |  |  |  |  |  |  |  |  |  |  |  |  |  |  | 1 |  |  |  |  |  |  |
| LO | 1 | Control measures humans |  |  |  |  |  |  |  |  |  |  |  |  |  |  |  |  |  |  |  | 1 |  |  |  |  |  |  |
| LO | 1 | Disease in human beyond control measures |  |  |  |  |  |  |  |  |  |  |  |  |  |  |  |  |  |  | 1 |  |  |  |  |  |  |  |
| LO | 1 | Human cause versus natural cause |  |  |  |  |  |  |  |  |  |  |  |  |  |  |  |  |  |  | 1 |  |  |  |  |  |  |  |
| LO | 1 | Potential to eradicate disease in animals |  |  |  |  |  |  |  |  |  |  |  |  |  |  |  |  |  |  | 1 |  |  |  |  |  |  |  |
| LO | 1 | Risk of bioterrorism |  |  |  |  |  |  |  |  |  |  |  |  |  |  |  |  |  |  | 1 |  |  |  |  |  |  |  |
| LO | 1 | Risk to food and water |  |  |  |  |  |  |  |  |  |  |  |  |  |  |  |  |  |  | 1 |  |  |  |  |  |  |  |
| LO | 1 | Surveillance in humans |  |  |  |  |  |  |  |  |  |  |  |  |  |  |  |  |  |  | 1 |  |  |  |  |  |  |  |
| LO | 1 | Vaccine/antiviral manufacturing time |  |  |  |  |  |  |  |  |  |  |  |  |  |  |  |  |  |  | 1 |  |  |  |  |  |  |  |
| LO | 1 | Visual cues to avoid disease in humans |  |  |  |  |  |  |  |  |  |  |  |  |  |  |  |  |  |  | 1 |  |  |  |  |  |  |  |
| LO | 1 | Counter measures |  |  |  |  |  |  |  |  |  |  | - | - | - | - |  |  | - | - |  |  | - | - | 1 | - | - |  |
| LO | 1 | Evidence assessment |  |  |  |  |  |  |  |  |  |  | - | - | - | - |  |  | - | - |  |  | - | - | 1 | - | - |  |
| LO | 1 | Resources |  |  |  |  |  |  |  |  |  |  | - | - | - | - |  |  | - | - |  |  | - | - | 1 | - | - |  |
| LO | 1 | reservoir or vector control |  |  |  |  |  |  |  |  |  |  |  |  |  |  |  |  |  | 1 |  |  |  |  |  |  | - |  |
| LO | 1 | Presence of a control plan |  |  |  |  |  |  |  |  |  |  |  |  |  |  |  |  |  | 1 |  |  |  |  |  |  | - |  |
| LO | 1 | Surveillance of pathogenic agent in region of interest |  |  |  |  |  |  |  |  |  |  |  |  |  |  |  |  |  | 1 |  |  |  |  |  |  | - |  |
| LO | 1 | PHLS added value |  | 1 | - |  | - |  |  |  |  |  |  | - |  |  |  |  |  |  |  |  |  |  |  |  |  |  |
| LO | 1 | Capacity for early detection via data pooling |  |  | 1 |  |  |  |  |  |  |  |  |  |  |  |  |  |  |  |  |  |  |  |  |  |  |  |
| LO | 1 | Capacity to provide extra awareness at national level |  |  | 1 |  |  |  |  |  |  |  |  |  |  |  |  |  |  |  |  |  |  |  |  |  |  |  |
| LO | 1 | Capacity to recognize threats requiring coordinated action |  |  | 1 |  |  |  |  |  |  |  |  |  |  |  |  |  |  |  |  |  |  |  |  |  |  |  |
| LO | 1 | Capacity to aid knowledge generation |  |  | 1 |  |  |  |  |  |  |  |  |  |  |  |  |  |  |  |  |  |  |  |  |  |  |  |
| LO | 1 | Improvement of programme evaluation |  |  | 1 |  |  |  |  |  |  |  |  |  |  |  |  |  |  |  |  |  |  |  |  |  |  |  |
| LO | 1 | knowledge advancement via pooling of resources |  |  | 1 |  |  |  |  |  |  |  |  |  |  |  |  |  |  |  |  |  |  |  |  |  |  |  |
| LO | 1 | Helps raise national standards |  |  | 1 |  |  |  |  |  |  |  |  |  |  |  |  |  |  |  |  |  |  |  |  |  |  |  |
| LO | 1 | Helps develop europe-wide surveillance and prevention |  |  | 1 |  |  |  |  |  |  |  |  |  |  |  |  |  |  |  |  |  |  |  |  |  |  |  |
| LO | 1 | Effectiveness of national and international surveillance |  |  |  |  |  |  |  |  |  |  |  |  |  | - |  |  |  | - |  |  |  | 1 | - |  | - |  |
| PH | 1 | Psychological impact in humans |  |  |  | - |  |  |  |  | - |  |  | - | - |  |  |  | - |  | 1 |  | - | - | - | - |  | PHC-02 |
| PH | 1 | Co-infection in humans |  |  |  | - |  |  |  |  |  |  |  | - | - | - |  |  | - |  | 1 |  | - | - | - | - |  | PHC-02 |
| PH | 1 | Public Health - degree of exposure |  |  |  | - |  |  |  |  |  |  | - | - | - | - |  |  | - | - |  |  | - | - | 1 | - | - | PHC-01 |
| PH | 1 | Likely incidence if introduced or re-emerged |  |  |  | - |  |  |  |  |  |  |  | - | - | - |  |  | - | - |  |  | - | 1 | - | - | - | REC-04 |
| RE | 1 | Occurrence in food / slaughter houses | - | - | - | - | - |  | - | - | - | - | - | 1 | - | - | - | - | - | 0 | - |  | - | - | - | - | - | REC-01 |
| RE | 1 | Long term effects on comm. Disease |  |  | - |  |  |  | 1 |  |  | - |  | - | - | - |  |  |  | 0 |  |  |  |  |  | - |  |  |
| RE | 1 | low incidence only maintained by PH act. |  |  | - |  |  |  | 1 |  |  | - |  | - | - | - |  |  |  | 0 |  |  |  |  |  | - |  |  |
| RE | 1 | Identification of reservoir or asymptomatic species |  |  | - |  |  |  |  |  |  |  | - | - | 1 | - |  |  | - | 0 |  |  | - | - | - | - | - |  |
| RE | 1 | Identification of vector species and existance of surveillance or study |  |  |  |  |  |  |  |  |  |  | - | - | 1 | - |  |  | - | 0 |  |  | - | - | - | - | - |  |
| RE | 1 | Impact of CC |  |  |  |  |  |  |  |  |  |  | - | - | 1 | - |  |  | - | 0 |  |  | - | - | - | - | - | REC-01 |
| RE | 1 | Ability of pathogen to mutate and adapt |  |  |  |  |  |  |  |  |  |  |  |  |  |  |  |  | - |  | 1 |  |  |  |  |  |  | REC-01 |
| RE | 1 | Combined disease risk and probability of infection |  |  |  |  |  |  |  |  |  |  |  |  |  |  |  |  | - |  | 1 |  |  |  |  |  |  | REC-01 |
| RE | 1 | Endemicity of disease due to CC |  |  |  |  |  |  |  |  |  |  |  |  |  |  |  |  | - |  | 1 |  |  |  |  |  |  | REC-01 |
| RE | 1 | Immunogenicity humans |  |  |  |  |  |  |  |  |  |  |  |  |  |  |  |  | - |  | 1 |  |  |  |  |  |  | REC-01 |
| RE | 1 | CC impact vectors and animal hosts |  |  |  |  |  |  |  |  |  |  |  |  |  |  |  |  | - |  | 1 |  |  |  |  |  |  | REC-01 |
| RE | 1 | Endemicity risk animals |  |  |  |  |  |  |  |  |  |  |  |  |  |  |  |  | - |  | 1 |  |  |  |  |  |  |  |
| RE | 1 | Endemicity risk humans |  |  |  |  |  |  |  |  |  |  |  |  |  |  |  |  | - |  | 1 |  |  |  |  |  |  | REC-02 |
| RE | 1 | Seasonality of disease |  |  |  |  |  |  |  |  |  |  |  |  |  |  |  |  | - |  | 1 |  |  |  |  |  |  | REC-01 |
| RE | 1 | Size of reservoir |  |  |  |  |  |  |  |  |  |  |  |  |  |  |  |  | - |  | 1 |  |  |  |  |  |  | REC-01 |
| RE | 1 | Geographical source of disease |  |  |  |  |  |  |  |  |  |  |  |  |  |  |  |  | - |  | 1 |  |  |  |  |  |  | REC-01 |
| RE | 1 | Evolutive characteristics of pathogen |  |  |  |  |  |  |  |  |  |  |  |  |  |  |  |  | - | 1 |  |  |  |  |  |  | - | REC-02 |
| RE | 1 | Presence/absence of vector/reservoir in region of interest |  |  |  |  |  |  |  |  |  |  |  |  |  |  |  |  | - | 1 |  |  |  |  |  |  | - | REC-01 |
| RE | 1 | Specificity of pathogen |  |  |  |  |  |  |  |  |  |  |  |  |  |  |  |  | - | 1 |  |  |  |  |  |  | - | REC-02 |
| RE | 1 | Persistence environment |  |  |  |  |  |  |  |  |  |  |  |  |  |  |  |  | - | 1 |  |  |  |  |  |  | - | REC-01 |
| RE | 1 | Current climatic conditions |  |  |  |  |  |  |  |  |  |  |  |  |  | - |  |  | - | - |  |  |  | 1 | - |  | - | REC-01 |
| RE | 1 | Geographic proximity |  |  |  |  |  |  |  |  |  |  |  |  |  | - |  |  | - | - |  |  |  | 1 | - |  | - | REC-01 |
| RE | 1 | Impact of annual temperature increase on pathogen |  |  |  |  |  |  |  |  |  |  |  |  |  | - |  |  | - | - |  |  |  | 1 | - |  | - | REC-01 |
| RE | 1 | Impact of summer ppt decrease on pathogen |  |  |  |  |  |  |  |  |  |  |  |  |  | - |  |  | - | - |  |  |  | 1 | - |  | - | REC-01 |
| RE | 1 | Impact of summer ppt increase on pathogen |  |  |  |  |  |  |  |  |  |  |  |  |  | - |  |  | - | - |  |  |  | 1 | - |  | - | REC-01 |
| RE | 1 | Impact of summer temperature decrease on pathogen |  |  |  |  |  |  |  |  |  |  |  |  |  | - |  |  | - | - |  |  |  | 1 | - |  | - | REC-01 |
| RE | 1 | Impact of summer temperature increase on pathogen |  |  |  |  |  |  |  |  |  |  |  |  |  | - |  |  | - | - |  |  |  | 1 | - |  | - | REC-01 |
| RE | 1 | Impact of winter ppt decrease on pathogen |  |  |  |  |  |  |  |  |  |  |  |  |  | - |  |  | - | - |  |  |  | 1 | - |  | - | REC-01 |
| RE | 1 | Impact of winter ppt increase on pathogen |  |  |  |  |  |  |  |  |  |  |  |  |  | - |  |  | - | - |  |  |  | 1 | - |  | - | REC-01 |
| RE | 1 | Impact of winter temperature decrease on pathogen |  |  |  |  |  |  |  |  |  |  |  |  |  | - |  |  | - | - |  |  |  | 1 | - |  | - | REC-01 |
| RE | 1 | Impact of winter temperature increase on pathogen |  |  |  |  |  |  |  |  |  |  |  |  |  | - |  |  | - | - |  |  |  | 1 | - |  | - | REC-01 |
| RE | 1 | Number of ways pathogen can enter region of interest |  |  |  |  |  |  |  |  |  |  |  |  |  | - |  |  | - | - |  |  |  | 1 | - |  | - | REC-01 |
| RE | 1 | Pathogenic taxonomic group |  |  |  |  |  |  |  |  |  |  |  |  |  | - |  |  | - | - |  |  | - | 1 | - |  | - | REC-01 |
| RE | 1 | Presence of definitive host species |  |  |  |  |  |  |  |  |  |  |  |  |  | - |  |  | - | - |  |  |  | 1 | - |  | - | REC-01 |
| RE | 1 | Presence of suitable vector in region of interest |  |  |  |  |  |  |  |  |  |  |  |  |  | - |  |  | - | - |  |  |  | 1 | - |  | - | REC-01 |
| RE | 1 | Type of climate pathogen can tolerate |  |  |  |  |  |  |  |  |  |  |  |  |  | - |  |  | - | - |  |  |  | 1 | - |  | - | REC-01 |
| SP | 1 | Discontent |  |  |  |  |  |  |  |  |  |  | - | - | - | - |  |  | - | - |  |  | - | - | 1 | - | - | SIC-02 |

PH – public health; LO – logistics; SP – Social perception; RE – Risk and epidemiology; AE – Animal and environmental health; EC – economics
