# Supplementary material for: Criteria for the prioritization of public health interventions for climate-sensitive vector-borne diseases in Quebec
Source: PLoS One. 2017 Dec 27;12(12):e0190049. doi: 10.1371/journal.pone.0190049 (PMC5744945; doi:10.1371/journal.pone.0190049)
Supplement: S2 Table — (DOCX) [file pone.0190049.s002.docx]

**S2 Table. Individual stakeholder weights for all criteria ordered by importance for the “Research” intervention domain**

| S1 | | S2 | | S3 | | S4 | | S5 | | S6 | | S7 | | S8 | | S9 | | S10 | |
| --- | --- | --- | --- | --- | --- | --- | --- | --- | --- | --- | --- | --- | --- | --- | --- | --- | --- | --- | --- |
| SOC-01 | 11 | SOC-03 | 15 | PHC-02 | 10 | PHC-02 | 15 | REC-01 | 10 | REC-01 | 9 | PHC-02 | 11 | SOC-01 | 16 | PHC-04 | 6 | PHC-01 | 28 |
| SOC-03 | 11 | PHC-02 | 12 | REC-02 | 10 | REC-02 | 10 | SOC-02 | 10 | REC-02 | 9 | PHC-01 | 9 | SOC-02 | 16 | REC-04 | 6 | REC-02 | 10 |
| REC-01 | 9 | PHC-01 | 9 | REC-03 | 10 | REC-04 | 10 | SOC-03 | 10 | PHC-02 | 9 | SOC-02 | 8 | SOC-03 | 10 | AEC-03 | 6 | REC-01 | 9 |
| REC-02 | 9 | PHC-03 | 9 | SIC-01 | 9 | SOC-03 | 10 | PHC-02 | 5 | PHC-01 | 8 | REC-02 | 7 | SOC-05 | 5 | ECC-03 | 6 | ECC-01 | 8 |
| REC-03 | 9 | REC-02 | 6 | SIC-02 | 6 | REC-01 | 5 | PHC-03 | 5 | REC-03 | 8 | PHC-03 | 6 | AEC-01 | 4 | SOC-04 | 6 | PHC-02 | 6 |
| PHC-01 | 6 | REC-03 | 6 | SOC-02 | 6 | PHC-03 | 5 | SIC-01 | 5 | AEC-03 | 7 | SIC-01 | 6 | AEC-02 | 4 | SOC-05 | 6 | ECC-02 | 5 |
| PHC-02 | 6 | ECC-01 | 5 | PHC-01 | 5 | REC-03 | 5 | SIC-02 | 5 | AEC-01 | 7 | SOC-01 | 6 | AEC-03 | 4 | REC-01 | 5 | PHC-03 | 4 |
| PHC-03 | 6 | ECC-02 | 5 | PHC-03 | 5 | AEC-02 | 5 | REC-04 | 5 | AEC-02 | 7 | REC-01 | 5 | REC-01 | 3 | PHC-03 | 5 | AEC-01 | 4 |
| SOC-02 | 4 | ECC-03 | 5 | PHC-04 | 5 | AEC-03 | 5 | AEC-01 | 5 | PHC-03 | 5 | PHC-04 | 4 | PHC-01 | 3 | SIC-02 | 5 | REC-04 | 4 |
| AEC-02 | 4 | REC-01 | 4 | AEC-03 | 5 | ECC-03 | 5 | AEC-02 | 5 | REC-04 | 5 | SIC-02 | 4 | PHC-02 | 3 | REC-02 | 5 | AEC-02 | 4 |
| SIC-01 | 4 | REC-04 | 4 | SOC-03 | 5 | SOC-01 | 5 | AEC-03 | 5 | SOC-03 | 5 | REC-03 | 4 | PHC-03 | 3 | REC-03 | 5 | SIC-02 | 3 |
| REC-04 | 3 | AEC-01 | 3 | ECC-01 | 4 | SIC-02 | 4 | ECC-01 | 5 | SOC-04 | 5 | REC-04 | 4 | PHC-04 | 3 | SOC-03 | 5 | ECC-03 | 3 |
| AEC-01 | 3 | AEC-02 | 3 | ECC-03 | 4 | SOC-02 | 4 | SOC-01 | 5 | PHC-04 | 4 | AEC-03 | 4 | SIC-01 | 3 | PHC-02 | 4 | REC-03 | 3 |
| AEC-03 | 3 | AEC-03 | 3 | REC-01 | 3 | PHC-01 | 3 | ECC-02 | 4 | ECC-01 | 3 | SOC-03 | 4 | SIC-02 | 3 | SIC-01 | 4 | AEC-03 | 3 |
| SOC-04 | 3 | SOC-04 | 3 | REC-04 | 3 | ECC-01 | 3 | PHC-04 | 3 | ECC-03 | 2 | AEC-02 | 4 | REC-02 | 3 | AEC-02 | 4 | SOC-03 | 3 |
| PHC-04 | 2 | SIC-01 | 3 | AEC-01 | 3 | PHC-04 | 2 | REC-02 | 3 | SOC-01 | 2 | ECC-01 | 4 | REC-03 | 3 | ECC-01 | 4 | PHC-04 | 2 |
| ECC-01 | 2 | SIC-02 | 3 | AEC-02 | 3 | ECC-02 | 2 | SOC-04 | 3 | SOC-02 | 2 | ECC-02 | 4 | REC-04 | 3 | ECC-02 | 4 | SIC-01 | 2 |
| ECC-02 | 2 | SOC-01 | 1 | ECC-02 | 2 | SIC-01 | 1 | PHC-01 | 2 | ECC-02 | 2 | ECC-03 | 3 | ECC-01 | 3 | SOC-01 | 4 | SOC-01 | 1 |
| SIC-02 | 2 | SOC-02 | 1 | SOC-01 | 2 | SOC-04 | 1 | REC-03 | 2 | SIC-01 | 2 | AEC-01 | 3 | ECC-02 | 3 | SOC-02 | 4 | SOC-05 | 1 |
| ECC-03 | 2 | PHC-04 | 0 | SOC-04 | 2 | AEC-01 | 0 | SOC-05 | 2 | SIC-02 | 2 | SOC-04 | 1 | SOC-04 | 3 | PHC-01 | 3 | SOC-02 | 1 |
| SOC-05 | 1 | SOC-05 | 0 | SOC-05 | 2 | SOC-05 | 0 | ECC-03 | 1 | SOC-05 | 2 | SOC-05 | 1 | ECC-03 | 2 | AEC-01 | 3 | SOC-04 | 0 |
|  | 100 |  | 100 |  | 100 |  | 100 |  | 100 |  | 100 |  | 100 |  | 100 |  | 100 |  | 100 |
