# Supplementary material for: Criteria for the prioritization of public health interventions for climate-sensitive vector-borne diseases in Quebec
Source: PLoS One. 2017 Dec 27;12(12):e0190049. doi: 10.1371/journal.pone.0190049 (PMC5744945; doi:10.1371/journal.pone.0190049)
Supplement: S6 Table — (DOCX) [file pone.0190049.s006.docx]

**S6 Table. Weight stability interval sensitivity analysis for all stakeholders for the prevention & control domain**

| Criteria | S1 | S2 | S3 | S4 | S5 | S6 | S7 | S8 | S9 | S10 |
| --- | --- | --- | --- | --- | --- | --- | --- | --- | --- | --- |
| PHC-01 | 5 (0-100) | 6 (0-100) | 5 (0-100) | 5 (0-100) | 8 (0-100) | 5 (0-100) | 11 (0-100) | 6 (1.5-100) | 5 (0-100) | 14 (0-100) |
| PHC-02 | 4(3-8) | 12 (7-25) | 10 (4-15) | 13 (11.5-23) | 2 (0-13) | 5 (0.5-18) | 12 (3.5-16) | 6 (2.5-9) | 4 (1.5-14) | 5 (0-7) |
| PHC-03 | 8(7-100) | 9 (8-100) | 5 (4.5-100) | 5 (3-11.5) | 5 (0-100) | 5 (4.5-100) | 5 (3-100) | 6 (1.5-10) | 6 (0-100) | 12 (0-100) |
| PHC-04 | 2 (0-100) | 3 (0-100) | 5 (0-100) | 2 (0-100) | 3 (0-100) | 4 (0-100) | 3 (0-100) | 2 (0-100) | 5 (0-100) | 4 (0-100) |
| SIC-01 | 3(2-23.5) | 5 (3.5-22) | 9 (8.5-15.5) | 1 (0-8) | 8 (0-20) | 4 (3.5-20) | 5 (3-16) | 10 (5-14) | 4 (0-16) | 2 (0-20) |
| SIC-02 | 3(0-23.5) | 0 (0-100) | 6 (0-13) | 4 (0-17) | 8 (0-20) | 4 (0-19) | 5 (0-16) | 10 (0-14) | 5 (0-17) | 3 (0-20) |
| REC-01 | 9(8-19) | 8 (7-21.5) | 3 (2.5-15) | 5 (3-10.5) | 10 (3.5-25) | 5 (4.5-18) | 6 (4.5-11) | 5 (1.5-8.5) | 6 (0-17) | 9 (0-11) |
| REC-02 | 8 (0-14) | 8 (0-16.5) | 10 (1.5-17) | 10 (0-17) | 1 (0-12) | 5 (0-13) | 9 (0-12) | 5 (0.5-16) | 5 (0-12) | 4 (0-5) |
| REC-03 | 8(1-8) | 8 (0-9) | 10 (2.5-10) | 5 (0-6.5) | 2 (0-8) | 5 (0-5) | 5 (2-6) | 5 (1-8) | 4 (0-9) | 2 (0.5-13) |
| REC-04 | 6(0-100) | 3 (0-100) | 3 (0-100) | 10 (0-100) | 2 (0-100) | 5 (0-100) | 5 (0-100) | 5 (0-100) | 5 (0-100) | 5 (0-100) |
| AEC-01 | 4(0-100) | 3 (0-100) | 3 (0-100) | 2 (0-100) | 5 (0-100) | 6 (0-100) | 2 (0-100) | 2 (0-100) | 4 (0-100) | 3 (0-100) |
| AEC-02 | 4(0-4) | 3 (0-5) | 3 (0-3) | 4 (0-5) | 5 (0-11) | 6 (0-6.5) | 2 (0-4) | 4 (0-7) | 4 (0-7) | 1 (0-14) |
| AEC-03 | 3(0-100) | 3 (0-100) | 5 (0-100) | 4 (0-100) | 5 (0-100) | 6 (0-100) | 2 (0-100) | 4 (0-100) | 5 (0-100) | 1 (0-100) |
| ECC-01 | 2(1-7) | 2 (0-100) | 4 (0-100) | 6 (4-100) | 8 (2.5-100) | 6 (0.5-100) | 2 (0-100) | 1 (0-100) | 4 (1-100) | 9 (0-100) |
| ECC-02 | 2(1-7) | 2 (0-100) | 2 (0-100) | 2 (0-100) | 5 (0-100) | 5 (0-100) | 2 (0-100) | 1 (0-100) | 4 (0-100) | 5 (0-100) |
| ECC-03 | 2(0-100) | 2 (0-100) | 4 (0-100) | 2 (0-100) | 2 (0-100) | 6 (0-100) | 2 (0-100) | 1 (0-100) | 5 (0-100) | 2 (0-100) |
| SOC-01 | 10 (0-100) | 3 (0-4) | 2 (0-2) | 2 (0-15) | 7 (1.5-13) | 5 (0-5) | 6 (0-7) | 12 (7.5-15.5) | 5 (2.5-11) | 4 (0-16) |
| SOC-02 | 9(0-9) | 13 (4-15) | 6 (0-6) | 15 (8.5-16) | 10 (0-17) | 5 (0-5.5) | 9 (6-11) | 12 (5-15) | 5 (0-9) | 8 (6.5-23.5) |
| SOC-03 | 6 (0-6) | 3 (0-8.5) | 5 (0-11) | 2 (0-4) | 2 (0-7) | 4 (0-9) | 5 (1-13.5) | 1 (0-5) | 5 (0-7) | 1 (0-12) |
| SOC-04 | 3 (0-100) | 6 (0-100) | 2 (0-100) | 1 (0-100) | 2 (0-100) | 3 (0-100) | 3 (0-100) | 1 (0-100) | 5 (0-100) | 5 (0-100) |
| SOC-05 | 2 (1-100) | 0 (0-100) | 2 (1.5-100) | 0 (0-100) | 1 (0-100) | 3 (2.5-100) | 3 (1-100) | 1 (0-5) | 5 (0-100) | 2 (0-100) |

S1-S10 – denotes stakeholders 1 through 10; Stakeholder assigned weights are given for all criteria followed by the stability interval in parentheses over which the ranking order for the 1^st^ position items is maintained. PHC – Public Health criteria; SIC – Social impact criteria; REC – Risk and epidemiology criteria; AEC – Animal and environmental health criteria; ECC - Economic criteria; SOC – Strategic and operational criteria
