# Supplementary material for: Criteria for the prioritization of public health interventions for climate-sensitive vector-borne diseases in Quebec
Source: PLoS One. 2017 Dec 27;12(12):e0190049. doi: 10.1371/journal.pone.0190049 (PMC5744945; doi:10.1371/journal.pone.0190049)
Supplement: S1 Appendix — (DOCX) [file pone.0190049.s007.docx]

# S1 Appendix. Supporting references used to assess disease scores for the pilot prioritization

Adam-Poupart, A., Smargiassi, A., Busque, M.-A., Duguay, P., Fournier, M., Zayed, J., Labrèche, F., 2014. Summer outdoor temperature and occupational heat-related illnesses in Quebec (Canada). Environ. Res. 134, 339–344. doi:10.1016/j.envres.2014.07.018

Aquino, M., Fyfe, M., MacDougall, L., Remple, V., 2004. West Nile virus in British Columbia. Emerg. Infect. Dis. 10, 1499 – 1501.

Artsob, H., Spence, L., 1991. Imported arbovirus infections in Canada 1974–89. Can. J. Infect. Dis. 2, 95–100.

Averett, E., Neuberger, J.S., Hansen, G., Fox, M.H., 2005. Evaluation of West Nile virus education campaign. Emerg. Infect. Dis. 11, 1751–1753.

Barber, L.M., Schleier III, J., Peterson, R.K.D., 2010. Economic cost analysis of west nile virus outbreak, sacramento county, california, USA, 2005. Emerg. Infect. Dis. 16, 480–486.

Berrang-Ford, L., McLean, J.D., Gyorkos, T.W., Ford, J.D., Ogden, N.H., 2009. Climate change and Malaria in Canada: a systems approach. Interdiscip. Perspect. Infect. Dis. 2009, 13.

Chartrand, A., Joncas, D., Fiset, M., Levac, É., Turgeon, N., 2015. Surveillance des maladies à déclaration obligatoire au Québec - Définitions nosologiques - Maladies d’origine infectieuse - 10e édition. Québec.

Dauphin, G., Zientara, S., Zeller, H., Murgue, B., 2004. West Nile: worldwide current situation in animals and humans. Comp. Immunol. Microbiol. Infect. Dis. 27, 343–355.

El Adlouni, S., Beaulieu, C., Ouarda, T., Gosselin, P., Saint-Hilaire, A., 2007. Effects of climate on West Nile Virus transmission risk used for public health decision-making in Quebec. Int. J. Health Geogr. 6, 40.

Elliott, S.J., Loeb, M., Harrington, D., Eyles, J., 2008. Heeding the Message? Determinants of Risk Behaviours for West Nile Virus. Can. J. Public Health Rev. Can. Santee Publique 99, 137–141. doi:10.2307/41995059

Elmieh, N., 2009. Public health responses to west nile virus: The role of risk perceptions and behavioral uncertainty in risk communication and policy.

Gould, L.H., Nelson, R.S., Griffith, K.S., Hayes, E.B., Piesman, J., Mead, P.S., 2008. Knowledge, attitudes, and behaviors regarding Lyme disease prevention among Connecticut residents, 1999–2004. Vector Borne Zoonotic Dis 8. doi:10.1089/vbz.2007.0221

Government of Canada, P.H.A. of C., 2014. Human Surveillance (2013) – Human West Nile Virus - Clinical Cases and Asymptomatic Infections in Canada - Public Health Agency of Canada [WWW Document]. URL http://www.phac-aspc.gc.ca/wnv-vwn/table/2013-2017-eng.php (accessed 4.9.15).

Gubler, D.J., 1998. Dengue and Dengue Hemorrhagic Fever. Clin. Microbiol. Rev. 11, 480–496.

Gujral, I.B., Zielinkski-Gutierrez, E.C., LeBailly, A., Nasci, R., 2007. Behavioral risks for west nile virus disease, Northern Colorado, 2003. Emerg. Infect. Dis. 13, 419–425.

Guzman, A., Istúriz, R.E., 2010. Update on the global spread of dengue. Int. J. Antimicrob. Agents 36, Supplement 1, S40 – S42. doi:http://dx.doi.org/10.1016/j.ijantimicag.2010.06.018

Halstead, S.B., 2007. Dengue. The Lancet 370, 1644 – 1652. doi:http://dx.doi.org/10.1016/S0140-6736(07)61687-0

Hayes, E.B., Gubler, D.J., 2006. West Nile Virus: epidemiology and clinical features of an emerging epidemic in the United States. Annu. Rev. Med. 57, 181–94.

Herrington, J.E., 2004. Risk perceptions regarding ticks and Lyme disease: a national survey. Am. J. Prev. Med. 26, 135–140. doi:10.1016/j.amepre.2003.10.010

Kramer, L.D., Styer, L.M., Ebel, G.D., 2008. A Global Perspective on the Epidemiology of West Nile Virus. Annu. Rev. Entomol. 53, 61–81. doi:10.1146/annurev.ento.53.103106.093258

Lanciotti, R.S., Kerst, A.J., Nasci, R.S., Godsey, M.S., Mitchell, C.J., Savage, H.M., Komar, N., Panella, N.A., Allen, B.C., Volpe, K.E., Davis, B.S., Roehrig, J.T., 2000. Rapid Detection of West Nile Virus from Human Clinical Specimens, Field-Collected Mosquitoes, and Avian Samples by a TaqMan Reverse Transcriptase-PCR Assay. J. Clin. Microbiol. 38, 4066–4071.

Locally Acquired Dengue --- Key West, Florida, 2009--2010, 2010. . Morb. Mortal. Wkly. Rep. MMWR 59, 577–581.

Love, S., Louis, D., Ellison, D. W., n.d. Greenfield’s Neuropathology, 8th ed.

McCarthy, T.A., Hadler, J.L., Julian, K., Walsh, S.J., Biggerstaff, B.J., Hinten, S.R., Baisley, C., Iton, A., Brennan, T., Nelson, R.S., Achambault, G., Marfin, A.A., Petersen, L.R., 2006. West Nile virus serosurvey and assessment of personal prevention efforts in an area with intense epizootic activity: Connecticut, 2000. Ann. N. Y. Acad. Sci. 951, 307–316.

Ogden, N.H., 2009. The emergence of Lyme disease in Canada. Can. Med. Assoc. J. 12, 1221–1224.

Pasvol, G., 2005. The treatment of complicated and severe malaria. Br. Med. Bull. 75-76, 29–47. doi:10.1093/bmb/ldh059

Petersen, L.R., 2015. West Nile Virus: From Africa to Europe, America, and Beyond, in: Sing, A. (Ed.), Zoonoses - Infections Affecting Humans and Animals. Springer Netherlands, pp. 937–975.

Petersen LR, Brault AC, Nasci RS, 2013. West nile virus: Review of the literature. JAMA-J. Am. Med. Assoc. 310, 308–315. doi:10.1001/jama.2013.8042

Public Health Agency of Canada, 2015. List of Nationally Notifiable Diseases [WWW Document]. URL http://dsol-smed.phac-aspc.gc.ca/dsol-smed/ndis/list-eng.php (accessed 12.2.15).

Ruiz, M.O., Tedesco, C., McTighe, T.J., Austin, C., Kitron, U., 2004. Environmental and social determinants of human risk during a West Nile virus outbreak in the greater Chicago area, 2002. Int. J. Health Geogr. 3, 8.

Sambri, V., Capobianchi, M., Charrel, R., Fyodorova, M., Gaibani, P., Gould, E., Niedrig, M., Papa, A., Pierro, A., Rossini, G., Varani, S., Vocale, C., Landini, M.P., 2013. West Nile virus in Europe: emergence, epidemiology, diagnosis, treatment, and prevention. Clin. Microbiol. Infect. 19, 699–704. doi:10.1111/1469-0691.12211

Swaroop, A., Jain, A., Kumhar, M., Parihar, N., Jain, S., 2007. Chikungunya fever. J. Indian Acad. Clin. Med. 8, 164–168.

Tuiten, W., Koenraadt, C.M., McComas, K., Harrington, L., 2009. The Effect of West Nile Virus Perceptions and Knowledge on Protective Behavior and Mosquito Breeding in Residential Yards in Upstate New York. EcoHealth 6, 42–51. doi:10.1007/s10393-009-0219-z

Wilson, S.D., Varia, M., Lior, L.Y., null, 2005. West Nile Virus: the buzz on Ottawa residents’ awareness, attitudes and practices. Can. J. Public Health Rev. Can. Sante Publique 96, 109–113.

World Health Organization, 2012. World Malaria Report 2012. Geneva, Switzerland.

World Health Organization, 2000. Severe falciparum malaria. Trans. R. Soc. Trop. Med. Hyg. 94, Supplement 1, 1 – 90. doi:http://dx.doi.org/10.1016/S0035-9203(00)90300-6

Zohrabian, A., Meltzer, M.I., Ratard, R., Billah, K., Molinari, N.A., Roy, K., 2004. West Nile Virus economic impact, Louisiana, 2002. Emerg. Infect. Dis. 10.

Zompi, S., Harris, E., 2012. Animal Models of Dengue Virus Infection. Viruses 4, 62–82. doi:10.3390/v4010062
